# Discontinuous Galerkin methods for nonlinear scalar hyperbolic conservation laws: divided difference estimates and accuracy enhancement

**DOI:** 10.1007/s00211-016-0833-y

**Published:** 2016-08-08

**Authors:** Xiong Meng, Jennifer K. Ryan

**Affiliations:** 1 School of Mathematics, University of East Anglia, Norwich, NR4 7TJ UK; 2 Department of Mathematics, Harbin Institute of Technology, Harbin, 150001 Heilongjiang China

**Keywords:** 65M60, 65M12, 65M15

## Abstract

In this paper, an analysis of the accuracy-enhancement for the discontinuous Galerkin (DG) method applied to one-dimensional scalar nonlinear hyperbolic conservation laws is carried out. This requires analyzing the divided difference of the errors for the DG solution. We therefore first prove that the $\alpha$-th order $(1 \le \alpha \le {k+1})$ divided difference of the DG error in the $L^2$ norm is of order ${k + \frac{3}{2} - \frac{\alpha }{2}}$ when upwind fluxes are used, under the condition that $|f'(u)|$ possesses a uniform positive lower bound. By the duality argument, we then derive superconvergence results of order ${2k + \frac{3}{2} - \frac{\alpha }{2}}$ in the negative-order norm, demonstrating that it is possible to extend the Smoothness-Increasing Accuracy-Conserving filter to nonlinear conservation laws to obtain at least $({\frac{3}{2}k+1})$th order superconvergence for post-processed solutions. As a by-product, for variable coefficient hyperbolic equations, we provide an explicit proof for optimal convergence results of order ${k+1}$ in the $L^2$ norm for the divided differences of DG errors and thus $({2k+1})$th order superconvergence in negative-order norm holds. Numerical experiments are given that confirm the theoretical results.

## Introduction

In this paper, we study the accuracy-enhancement of semi-discrete discontinuous Galerkin (DG) methods for solving one-dimensional scalar conservation laws

1.1a $$\begin{aligned} u_t + f(u)_x = 0,\quad (x,t)\in (a, b) \times (0, T], \end{aligned}$$

1.1b $$\begin{aligned} u(x,0) = u_0(x), \quad x \in \Omega = (a, b), \end{aligned}$$

where $u_0(x)$ is a given smooth function. We assume that the nonlinear flux function *f*(*u*) is sufficiently smooth with respect to the variable *u* and the exact solution is also smooth. For the sake of simplicity and ease in presentation, we only consider periodic boundary conditions. We show that the $\alpha$-th order $(1 \le \alpha \le {k+1})$ divided difference of the DG error in the $L^2$ norm achieves $({k + \frac{3}{2} - \frac{\alpha }{2}})$th order when upwind fluxes are used, under the condition that $|f'(u)|$ possesses a uniform positive lower bound. By using a duality argument, we then derive superconvergence results of order ${2k + \frac{3}{2} - \frac{\alpha }{2}}$ in the negative-order norm. This allows us to demonstrate that it is possible to extend the post-processing technique to nonlinear conservation laws to obtain at least $({\frac{3}{2}k+1})$th order of accuracy. In addition, for variable coefficient hyperbolic equations that have been discussed in [19], we provide an explicit proof for optimal error estimates of order ${k+1}$ in the $L^2$ norm for the divided differences of the DG errors and thus ${2k+1}$ in the negative-order norm.

Various superconvergence properties of DG methods have been studied in the past two decades, which not only provide a deeper understanding about DG solutions but are useful for practitioners. According to different measurements of the error, the superconvergence of DG methods is mainly divided into three categories. The first one is superconvergence of the DG error at Radau points, which is typically measured in the discrete $L^2$ norm and is useful to resolve waves. The second one is superconvergence of the DG solution towards a particular projection of the exact solution (supercloseness) measured in the standard $L^2$ norm, which lays a firm theoretical foundation for the excellent behaviour of DG methods for long-time simulations as well as adaptive computations. The last one is the superconvergence of post-processed solution by establishing negative-order norm error estimates, which enables us to obtain a higher order approximation by simply post-processing the DG solution with a specially designed kernel at the very end of the computation. In what follows, we shall review some superconvergence results for the aforementioned three properties and restrict ourselves to hyperbolic equations to save space. For superconvergence of DG methods for other types of PDEs, we refer to [21].

Let us briefly mention some superconvergence results related to the Radau points and supercloseness of DG methods for hyperbolic equations. Adjerid and Baccouch [1–3] studied the superconvergence property as well as the a posteriori error estimates of the DG methods for one- and two-dimensional linear steady-state hyperbolic equations, in which superconvergence of order $k+2$ and ${2k+1}$ are obtained at downwind-biased Radau points and downwind points, respectively. Here and below, *k* is the highest polynomial degree of the discontinuous finite element space. For time-dependent linear hyperbolic equations, Cheng and Shu [9] investigated supercloseness for linear hyperbolic equations, and they obtained superconvergence of order $k+\frac{3}{2}$ towards a particular projection of the exact solution, by virtue of construction and analysis of the so-called generalized slopes. Later, by using a duality argument, Yang and Shu [24] proved superconvergence results of order $k+2$ of the DG error at downwind-biased points as well as cell averages, which imply a sharp $(k+2)$th order supercloseness result. By constructing a special correction function and choosing a suitable initial discretization, Cao et al. [7] established a supercloseness result towards a newly designed interpolation function. Further, based on this supercloseness result, for the DG error they proved the $({2k+1})$th order superconvergence at the downwind points as well as domain average, $(k+2)$-th order superconvergence at the downwind-biased Radau points, and superconvergent rate ${k+1}$ for the derivative at interior Radau points. We would like to remark that the work of [7, 24] somewhat indicates the possible link between supercloseness and superconvergence at Radau points. For time-dependent nonlinear hyperbolic equations, Meng et al. [18] proved a supercloseness result of order $k+\frac{3}{2}$. For superconvergent posteriori error estimates of spatial derivative of DG error for nonlinear hyperbolic equations, see [4].

Let us now mention in particular some superconvergence results of DG methods regarding negative-order norm estimates and post-processing for hyperbolic equations. The basic idea of post-processing is to convolve the numerical solution by a local averaging operator with the goal of obtaining a better approximation and typically of a higher order. Motivated by the work of Bramble and Schatz in the framework of continuous Galerkin methods for elliptic equations [5], Cockburn et al. [11] established the theory of post-processing techniques for DG methods for hyperbolic equations by the aid of negative-order norm estimates. The extension of this post-processing technique was later fully studied by Ryan et al. in different aspects of problems, e.g. for general boundary condition [20], for nonuniform meshes [13] and for applications in improving the visualization of streamlines [22] in which it is labeled as a Smoothness-Increasing Accuracy-Conserving (SIAC) filter. For the extension of the SIAC filter to linear convection-diffusion equations, see [15].

By the post-processing theory [5, 11], it is well known that negative-order norm estimates of divided differences of the DG error are important tools to derive superconvergent error estimates of the post-processed solution in the $L^2$ norm. Note that for purely linear equations [11, 15], once negative-order norm estimates of the DG error itself are obtained, they trivially imply negative-order norm estimates for the divided differences of the DG error. However, the above framework is no longer valid for variable coefficient or nonlinear equations. In this case, in order to derive superconvergent estimates about the post-processed solution, both the $L^2$ norm and negative-order norm error estimates of divided differences should be established. In particular, for variable coefficient hyperbolic equations, although negative-order norm error estimates of divided differences are given in [19], the corresponding $L^2$ norm estimates are not provided. For nonlinear hyperbolic conservation laws, Ji et al. [16] showed negative-order norm estimates for the DG error itself, leaving the estimates of divided differences for future work.

For nonlinear hyperbolic equations under consideration in this paper, it is therefore important and interesting to address the above issues by establishing both the $L^2$ norm and negative-order norm error estimates for the divided differences. The major part of this paper is to show $L^2$ norm error estimates for divided differences, which are helpful for us to obtain a higher order of accuracy in the negative-order norm and thus the superconvergence of the post-processed solutions. We remark that it requires $|f'(u)|$ having a uniform positive lower bound due to the technicality of the proof. The generalization from purely linear problems [11, 15] to nonlinear hyperbolic equations in this paper involves several technical difficulties. One of these is how to establish important relations between the spatial derivatives and time derivatives of a particular projection of divided differences of DG errors. Even if the spatial derivatives of the error are switched to their time derivatives, it is still difficult to analyze the time derivatives of the error; for more details, see Sect. 3.2 and also the appendix. Another important technicality is how to construct a suitable dual problem for the divided difference of the nonlinear hyperbolic equations. However, it seems that it is not trivial for the two-dimensional extension, especially for establishing the relations between spatial derivatives and time derivatives of the errors. The main tool employed in deriving $L^2$ norm error estimates for the divided differences is an energy analysis. To deal with the nonlinearity of the flux functions, Taylor expansion is used following a standard technique in error estimates for nonlinear problems [25]. We would like to remark that the superconvergence analysis in this paper indicates a possible link between supercloseness and negative-order norm estimates.

This paper is organized as follows. In Sect. 2, we give the DG scheme for divided differences of nonlinear hyperbolic equations, and present some preliminaries about the discontinuous finite element space. In Sect. 3, we state and discuss the $L^2$ norm error estimates for divided differences of nonlinear hyperbolic equations, and then display the main proofs followed by discussion of variable coefficient hyperbolic equations. Section 4 is devoted to the accuracy-enhancement superconvergence analysis based on negative-order norm error estimates of divided differences. In Sect. 5, numerical experiments are shown to demonstrate the theoretical results. Concluding remarks and comments on future work are given in Sect. 6. Finally, in the appendix we provide the proofs for some of the more technical lemmas.

## The DG scheme and some preliminaries

### The DG scheme

In this section, we follow [11, 12] and present the DG scheme for divided differences of the problem (1.1).

Let $a = x_\frac{1}{2}< x_\frac{3}{2}< \cdots < x_{N+\frac{1}{2}} = b$ be a partition of $\Omega = (a, b)$, and set $x_j = (x_{j-\frac{1}{2}} + x_{j+\frac{1}{2}})/2$. Since we are focused on error analysis of both the $L^2$ norm and the negative-order norm for divided differences of the DG solution and the problem under consideration is assumed to be periodic, we shall introduce two overlapping uniform (translation invariant) meshes for $\Omega$, namely $I_j = (x_{j-\frac{1}{2}},x_{j+\frac{1}{2}})$ and $I_{j+ \frac{1}{2}}= (x_j, x_{j+1})$ with mesh size $h = x_{j+\frac{1}{2}} - x_{j-\frac{1}{2}}$. Associated with these meshes, we define the discontinuous finite element space

$$\begin{aligned} { V_{h}^\alpha } = {\left\{ v : v|_{I_{j'}} \in P^{k}(I_{j'}),\quad \forall j' = j + \frac{\ell }{2}, \ell = \alpha \mod 2,\quad j = 1, \ldots , N\right\} ,} \end{aligned}$$

where $P^{k}(I_{j'})$ denotes the set of polynomials of degree up to *k* defined on the cell $I_{j'}:= (x_{j' - \frac{1}{2}}, x_{j' + \frac{1}{2}})$. Here and below, $\alpha$ represents the $\alpha$-th order divided difference of a given function, whose definition is given in (2.6a). Clearly, ${ V_{h}^\alpha }$ is a piecewise polynomial space on mesh $I_{j'}= I_j$ for even $\alpha$ (including $\alpha = 0$) and a piecewise polynomial space on mesh $I_{j'}= I_{j+\frac{1}{2}}$ for odd $\alpha$ of the DG scheme. For simplicity, for even $\alpha$, we would like to use $V_h$ to denote the standard finite element space of degree *k* defined on the cell $I_j$, if there is no confusion. Since functions in ${ V_{h}^\alpha }$ may have discontinuities across element interfaces, we denote by $w_i^-$ and $w_i^+$ the values of *w*(*x*) at the discontinuity point $x_i$ from the left cell and the right cell, respectively. Moreover, we use $[\![{w}]\!] = w^+ - w^-$ and $\{\!\{{w}\}\!\} = \frac{1}{2}(w^+ + w^-)$ to represent the jump and the mean of *w*(*x*) at each element boundary point.

The $\alpha$-th order divided difference of the nonlinear hyperbolic conservation law is

2.1a $$\begin{aligned}&\partial _h^{\alpha } u_t + \partial _h^{\alpha } f(u)_x = 0,\quad (x,t)\in {\Omega ^\alpha } \times (0, T],\end{aligned}$$

2.1b $$\begin{aligned}&\partial _h^{\alpha } u(x,0) = \partial _h^{\alpha } u_0(x), \quad x \in {\Omega ^\alpha }, \end{aligned}$$

where ${\Omega ^\alpha } = (a + \frac{\ell }{2} h, b+ \frac{\ell }{2} h )$ with $\ell = \alpha \mod 2$. Clearly, (2.1) reduces to (1.1) when $\alpha = 0$. Then the approximation of the semi-discrete DG method for solving (2.1) becomes: find the unique function $u_h = u_h(t) \in V_h$ (and thus $\partial _h^{\alpha } u_h \in { V_{h}^\alpha }$) such that the *weak* formulation

2.2 $$\begin{aligned} ((\partial _h^\alpha u_h)_t, v_h)_{j'} = {\mathcal H}_{j'}(\partial _h^\alpha f(u_h), v_h) \end{aligned}$$

holds for all $v_h \in { V_{h}^\alpha }$ and all $j = 1, \ldots , N.$ Note that, by (2.6a), for any $x \in I_{j'}$ and *t*, $\partial _h^{\alpha } u_h(x,t)$ is a linear combination of the values of $u_h$ at $\alpha +1$ equally spaced points of length *h*, namely $x - \frac{\alpha }{2} h, \ldots , x+ \frac{\alpha }{2} h$. Here and in what follows, $\left( \cdot ,\cdot \right) _{j'}$ denotes the usual inner product in $L^2(I_{j'})$, and ${{\mathcal {H}}}_{j'}\left( \cdot ,\cdot \right)$ is the DG spatial discretization operator defined on each cell $I_{j'}= (x_{j' - \frac{1}{2}}, x_{j' + \frac{1}{2}})$, namely

$$\begin{aligned} {{\mathcal {H}}}_{j'}\left( w,v\right) = \left( w,v_x\right) _{j'} - \hat{w} v^-\left| _{j' + \frac{1}{2}} + \hat{w} v^+\right| _{j' - \frac{1}{2}}. \end{aligned}$$

We point out that in order to obtain a useful bound for the $L^2$ norm error estimates of divided differences, the numerical flux $\hat{f}_{j+\frac{1}{2}}$ is chosen to be an upwind flux, for example, the well-known Godunov flux. Moreover, the analysis requires a condition that $|f'(u)|$ has a uniform positive lower bound. Without loss of generality, throughout the paper, we only consider $f'(u) \ge \delta >0$, and thus $\hat{w} = w^-$. Therefore,

2.3a $$\begin{aligned} {{\mathcal {H}}}_{j'}\left( w,v\right)&= \left( w,v_x\right) _{j'} - w^- v^-|_{j' + \frac{1}{2}} + w^- v^+|_{j' - \frac{1}{2}}\end{aligned}$$

2.3b $$\begin{aligned}&= - \left( w_x,v\right) _{j'} - ([\![{w}]\!] v^+)_{j' - \frac{1}{2}}. \end{aligned}$$

For periodic boundary conditions under consideration in this paper, we use ${{\mathcal {H}}}$ to denote the summation of ${{\mathcal {H}}}_{j'}$ with respect to cell $I_{j'}$, that is

2.4a $$\begin{aligned} {{\mathcal {H}}} (w,v)&= \left( w,v_x\right) + {\sum _{j = 1}^N}(w^- [\![{v}]\!])_{j' + \frac{1}{2}}\end{aligned}$$

2.4b $$\begin{aligned}&= - \left( w_x,v\right) - {\sum _{j = 1}^N}([\![{w}]\!] v^+)_{j' - \frac{1}{2}}, \end{aligned}$$

where $\left( w,v\right) = {\sum _{j = 1}^N}\left( w,v\right) _{j'}$ represents the inner product in $L^2({\Omega ^\alpha })$. Note that we have used the summation with respect to *j* instead of $j'$ to distinguish two overlapping meshes, since $j' = j$ for even $\alpha$ and $j' = j+\frac{1}{2}$ for odd $\alpha$.

### Preliminaries

We will adopt the following convention for different constants. We denote by *C* a positive constant independent of *h* but may depend on the exact solution of the Eq. (2.1), which could have a different value in each occurrence. To emphasize the nonlinearity of the flux *f*(*u*), we employ ${C_{\star }}$ to denote a nonnegative constant depending solely on the maximum of a high order derivative $|f^{m}|$ ($m \ge 2$). We remark that ${C_{\star }}= 0$ for a linear flux function $f(u) = cu$ or a variable coefficient flux function $f(u) = a(x) u$, where *c* is a constant and *a*(*x*) is a given smooth function.

Prior to analyzing the $L^2$ norm and the negative-order norm error estimates of divided differences, let us present some notation, definitions, properties of DG discretization operator, and basic properties about SIAC filters. These preliminary results will be used later in the proof of superconvergence property.

#### Sobolev spaces and norms

We adopt standard notation for Sobolev spaces. For any integer $s \ge 0$, we denote by $W^{s,p}(D)$ the Sobolev space on subdomain $D \subset \Omega$ equipped with the norm ${\Vert {\cdot }\Vert }_{s,p,D}$. In particular, if $p = 2$, we set $W^{s,p}(D) = H^s(D)$, and ${\Vert {\cdot }\Vert }_{s,p,D} = {\Vert {\cdot }\Vert }_{s,D}$, and further if $s = 0$, we set ${\Vert {\cdot }\Vert }_{s,D} = {\Vert {\cdot }\Vert }_D$. Throughout the paper, when $D = \Omega$, we will omit the index *D* for convenience. Furthermore, the norms of the *broken* Sobolev spaces $W^{s,p}(\Omega _{h}) := \{ u \in L^2 (\Omega ) :u|_{D} \in W^{s,p}(D), ~\forall D \subset \Omega \}$ with $\Omega _{h}$ being the union of all cells can be defined analogously. The Bochner space can also be easily defined. For example, the space $L^1([0,T];H^s(D))$ is equipped with the norm ${\Vert {\cdot }\Vert }_{L^1([0,T];H^s(D))} = {\int _{0}^T{{\Vert {\cdot }\Vert }_{s, D}}dt }$.

Additionally, we denote by ${\Vert {\cdot }\Vert _{\Gamma _{\!h}}}$ the standard $L^2$ norm on the cell interfaces of the mesh $I_{j'}$. Specifically, for the one-dimensional case under consideration in this paper, ${\Vert {v}\Vert _{\Gamma _{\!h}}^2} = {\sum _{j = 1}^N}{\Vert {v}\Vert }_{\partial I_{j'}}^2$ with ${\Vert {v}\Vert }_{\partial I_{j'}} = {( (v_{j'-1/2}^+)^2 + (v_{j'+1/2}^-)^2)}^\frac{1}{2}$. To simplify notation in our later analysis, following [23], we would like to introduce the so-called *jump seminorm* ${\left| [{v}]\right| } = ({\sum _{j = 1}^N}{[\![{v}]\!]}_{j' - \frac{1}{2}}^2)^\frac{1}{2}$ for $v \in H^1(\Omega _{h})$.

In the post-processing framework, it is useful to consider the negative-order norm, defined as: Given $\ell > 0$ and domain $\Omega$,

2.5 $$\begin{aligned} \Vert {v}\Vert _{-\ell ,\Omega } = \sup _{\Phi \in C_0^\infty (\Omega )} \frac{\left( v,\Phi \right) }{{\Vert {\Phi }\Vert }_{\ell }}. \end{aligned}$$

#### Properties for divided differences

For any function *w* and integer $\gamma$, the following standard notation of *central* divided difference is used

2.6a $$\begin{aligned} \partial _h^{\gamma } w(x) = \frac{1}{h^\gamma } \sum _{i=0}^\gamma (-1)^i {\left( {\begin{array}{c}\gamma \\ i\end{array}}\right) } w \left( x + \left( \frac{\gamma }{2} - i \right) h\right) . \end{aligned}$$

Note that the above notation is still valid even if *w* is a piecewise function with possible discontinuities at cell interfaces. In later analysis, we will repeatedly use the properties about divided differences, which are given as follows. For any functions *w* and *v*

2.6b $$\begin{aligned} \partial _h^{\gamma } \left( w(x)v(x)\right) = \sum _{i=0}^\gamma {\left( {\begin{array}{c}\gamma \\ i\end{array}}\right) } \partial _h^{i} w \left( x + \frac{\gamma -i}{2} h \right) \partial _h^{\gamma -i} v \left( x - \frac{i}{2} h \right) , \end{aligned}$$

which is the so-called Leibniz rule for the divided difference. Moreover, for sufficiently smooth functions *w*(*x*), by using Taylor expansion with integral form of the remainder, we can easily verify that $\partial _h^{\gamma } w$ is a second order approximation to $\partial _{x}^{\gamma }w$, namely

2.6c $$\begin{aligned} \partial _h^{\gamma } w(x) = \partial _{x}^{\gamma }{w(x)} + C_\gamma \, h^2 \psi _\gamma (x), \end{aligned}$$

where $C_\gamma$ is a positive constant and $\psi _\gamma$ is a smooth function; for example, $C_\gamma = 1/8, 1, 3/32$ for $\gamma = 1,2,3$, and

$$\begin{aligned} \psi _\gamma (x) = \frac{1}{(\gamma + 1)!} {\int _{0}^1{\left( \partial _{x}^{\gamma + 2}w \left( x+ \frac{\gamma }{2} h s \right) + \partial _{x}^{\gamma + 2}w \left( x- \frac{\gamma }{2} hs \right) \right) (1 -s )^{\gamma +1} }\,ds }. \end{aligned}$$

Here and below, $\partial _{x}^{\gamma }(\cdot )$ denotes the $\gamma$-th order partial derivative of a function with respect to the variable *x*; likewise for $\partial _{t}^{\gamma }(\cdot )$. The last identity is the so-called summation by parts, namely

2.6d $$\begin{aligned} \left( \partial _h^{\gamma } w(x),v(x)\right) = (-1)^\gamma \left( w(x),\partial _h^{\gamma } v(x)\right) . \end{aligned}$$

In addition to the properties of divided differences for a single function *w*(*x*), the properties of divided differences for a composition of two or more functions are also needed. However, we would like to emphasize that properties (2.6a), (2.6b), (2.6d) are always valid whether *w* is a single function or *w* is a composition of two or more functions. As an extension from the single function case in (2.6c) to the composite function case, the following property (2.6e) is subtle, it requires a more delicate argument for composite functions. Without loss of generality, if *w* is the composition of two smooth functions *r* and *u*, i.e., $w(x) := r (u(x))$, we can prove the following identity

2.6e $$\begin{aligned} \partial _h^{\gamma } r(u(x)) = \partial _{x}^{\gamma }r(u(x)) + C_\gamma \, h \, \Psi _{\!\gamma }(x), \end{aligned}$$

where $C_\gamma$ is a positive constant and $\Psi _\gamma$ is a smooth function. We can see that, unlike (2.6c), the divided difference of a composite function is a first order approximation to its derivative of the same order. This finding, however, is sufficient in our analysis; see Corollary 1.

It is worth pointing out that in (2.6e), $\partial _{x}^{\gamma }r (u(x))$ and $\partial _h^{\gamma } r (u(x))$ should be understood in the sense of the chain rule for high order derivatives and divided differences of composite functions, respectively. In what follows, we use $f[x_0, \ldots , x_\gamma ]$ to denote the standard $\gamma$-th order Newton divided difference, that is

$$\begin{aligned} f[x_\nu ]&:= f(x_\nu ), \quad 0 \le \nu \le \gamma , \\ f[x_\nu , \ldots , x_{\nu + \mu }]&:= \frac{f[x_{\nu +1}, \ldots , x_{\nu + \mu }] - f[x_{\nu }, \ldots , x_{\nu + \mu - 1}]}{x_{\nu + \mu } - x_\nu }, \\&\quad \quad 0 \le \nu \le \gamma - \mu , \quad 1 \le \mu \le \gamma . \end{aligned}$$

It is easy to verify that

2.7 $$\begin{aligned} \partial _h^{\gamma } r(u(x)) = \gamma ! \, r[x_0, \ldots , x_\gamma ], \end{aligned}$$

where $$x_i = x + \frac{2 i - \gamma }{2} h$$ $$(0 \le i \le \gamma )$$.

For completeness, we shall list the chain rule for the derivatives (the well-known Faà di Bruno’s Formula) and also for the divided differences [14]; it reads

$$\begin{aligned} \partial _{x}^{\gamma }r(u(x))&= \sum \frac{\gamma !}{b_1 ! \cdots b_\gamma !} r^{(\ell )}(u(x)) \left( \frac{\partial _{x} u(x)}{1 !}\right) ^{b_1} \cdots \left( \frac{\partial _{x}^{\gamma }u(x)}{\gamma !}\right) ^{b_\gamma }, \\ r[x_0, \ldots , x_\gamma ]&= \sum _{\ell = 1}^\gamma r[u_0, \ldots , u_\ell ] \, A_{\ell , \gamma } u, \end{aligned}$$

where $u_i = u(x_i)$, and the sum is over all $\ell = 1, \ldots , \gamma$ and non-negative integer solutions $b_1,\ldots , b_\gamma$ to

$$\begin{aligned} b_1 + 2 b_2 + \cdots + \gamma b_\gamma = \gamma , \quad b_1 + \cdots + b_\gamma = \ell , \end{aligned}$$

and

$$\begin{aligned} A_{\ell , \gamma } u = \sum _{\ell = \alpha _0 \le \alpha _1 \le \cdots \le \alpha _\ell = \gamma } \prod _{\beta = 0}^{\ell - 1} u[x_\beta , x_{\alpha _\beta },\ldots ,x_{\alpha _{\beta +1}}] \end{aligned}$$

with the sum being over integers $\alpha _1, \ldots , \alpha _{\ell - 1 }$ such that $\ell \le \alpha _1 \le \cdots \le \alpha _{\ell - 1} \le \gamma$.

It follows from the mean value theorem for divided differences that

$$\begin{aligned} \lim _{h \rightarrow 0} r[x_0, \ldots , x_\gamma ] = \frac{\partial _{x}^{\gamma }r(u(x))}{\gamma !}. \end{aligned}$$

Consequently, by (2.7),

$$\begin{aligned} \lim _{h \rightarrow 0} \partial _h^{\gamma } r(u(x)) = \partial _{x}^{\gamma }r(u(x)). \end{aligned}$$

We are now ready to prove (2.6e) for the relation between the divided difference and the derivative of composite functions. Using a similar argument as that in the proof of (2.6c) for $A_{\ell , \gamma } u$ and the relation that

$$\begin{aligned} r[u_0,\ldots ,u_\gamma ] = \frac{r^{(\gamma )}(u_{\frac{\gamma }{2}})}{\gamma !} + C_\gamma \, h \, \psi (u_0,u_1,\ldots ,u_\gamma ), \end{aligned}$$

due to the smoothness of $u_i$ and the fact that $u_i$ may not necessarily be equally spaced, with $u_{\frac{\gamma }{2}} = u(x)$ and $\psi (u_0,u_1,\ldots ,u_\gamma )$ being smooth functions, we can obtain the relation (2.6e).

#### The inverse and projection properties

Now we list some inverse properties of the finite element space ${ V_{h}^\alpha }$. For any $p \in { V_{h}^\alpha }$, there exists a positive constant *C* independent of *p* and *h*, such that

$$\begin{aligned} {(i) }~~{\Vert {\partial _x p}\Vert } \le C h^{-1} {\Vert {p}\Vert }; \quad {(ii) }~~{\Vert {p}\Vert _{\Gamma _{\!h}}} \le C h^{-1/2} {\Vert {p}\Vert }; \quad {(iii) }~~{\Vert {p}\Vert _{\infty }} \le C h^{-1/2} {\Vert {p}\Vert }. \end{aligned}$$

Next, we introduce the standard $L^2$ projection of a function $q \in L^2(\Omega )$ into the finite element space $V_h^k$, denoted by $P_k q$, which is a unique function in $V_h^k$ satisfying

2.8 $$\begin{aligned} \left( q - P_k q,v_h\right) = 0,\quad \forall v_h \in V_h^k. \end{aligned}$$

Note that the proof of accuracy-enhancement of DG solutions for linear equations relies only on an $L^2$ projection of the initial condition [11, 15]. However, for variable coefficient and nonlinear hyperbolic equations, a suitable choice of the initial condition and a superconvergence relation between the spatial derivative and time derivative of a particular projection of the error should be established, since both the $L^2$ norm and negative-order norm error estimates of divided differences need to be analyzed. In what follows, we recall two kinds of Gauss–Radau projections $P_h^\pm$ into $V_h$ following a standard technique in DG analysis [8, 25]. For any given function $q \in H^1(\Omega _h)$ and an arbitrary element $I_{j'}=(x_{j'-\frac{1}{2}},x_{j'+\frac{1}{2}}),$ the special Gauss–Radau projection of *q*, denoted by $P_h^\pm q$, is the unique function in $V_h^k$ satisfying, for each $j'$,

2.9a $$\begin{aligned}&(q - P_h^+q, v_h)_{j'} = 0,\quad \forall v_h \in P^{k-1}(I_{j'}), \quad (q - P_h^+q)^{+}_{j'-\frac{1}{2}} =0; \end{aligned}$$

2.9b $$\begin{aligned}&(q - P_h^-q, v_h)_{j'}= 0,\quad \forall v_h \in P^{k-1}(I_{j'}), \quad (q - P_h^-q)^{-}_{j'+\frac{1}{2}} =0. \end{aligned}$$

We would like to remark that the exact collocation at one of the end points on each cell plus the orthogonality property for polynomials of degree up to $k - 1$ of the Gauss–Radau projections $P^\pm _h$ play an important role and are used repeatedly in the proof. We denote by ${\eta }= q(x) - {{{\mathbb {Q}}}_h} q(x)~( {{{\mathbb {Q}}}_h} = P_k$ or $P^\pm _h )$ the projection error, then by a standard scaling argument [6, 10], it is easy to obtain, for smooth enough *q*(*x*), that,

2.10a $$\begin{aligned} {\Vert {{\eta }}\Vert } + h {\Vert {{{\eta }}_x}\Vert } + h^{1/2} {\Vert {{\eta }}\Vert _{\Gamma _{\!h}}} \le Ch^{k+1} \Vert {q}\Vert _{{k+1}}. \end{aligned}$$

Moreover,

2.10b $$\begin{aligned} {\Vert {{\eta }}\Vert _{\infty }}\le C h^{k+1}{\Vert {q}\Vert }_{k+1,\infty }. \end{aligned}$$

#### The properties of the DG spatial discretization

To perform the $L^2$ error estimates of divided differences, several properties of the DG operator ${{\mathcal {H}}}$ are helpful, which are used repeatedly in our proof; see Sect. 3.

##### Lemma 1

Suppose that *r*(*u*(*x*, *t*)) $($ $r = f'(u), \partial _{t} f'(u)$ etc) is smooth with respect to each variable. Then, for any $w, v \in { V_{h}^\alpha }$, there holds the following inequality

2.11a $$\begin{aligned} {{\mathcal {H}}} (rw,v) \le {C_{\star }}\left( {\Vert {w}\Vert } + {\Vert {w_x}\Vert } + h^{-\frac{1}{2}}{\left| [{w}]\right| }\right) {\Vert {v}\Vert }, \end{aligned}$$

and in particular, if $r = f'(u)\ge \delta > 0$, there holds2.11b


##### Proof

Let us first prove (2.11b), which is a straightforward consequence of the definition of ${{\mathcal {H}}}$, since, after a simple integration by partsWe would like to emphasize that (2.11b) is still valid even if the smooth function *r* and $w \in { V_{h}^\alpha }$ depend on different *x*, e.g. $x, x+\frac{h}{2}$ etc, as only integration by parts as well as the boundedness of *r* is used here.

To prove (2.11a), we consider the equivalent *strong* form of ${{\mathcal {H}}}$ (2.4b). An application of Cauchy–Schwarz inequality and inverse inequality (ii) leads to

$$\begin{aligned} {{\mathcal {H}}} (rw,v)&= - \left( r_x w,v\right) - \left( r w_x,v\right) - {\sum _{j = 1}^N}(r [\![{w}]\!] v^+ )_{j'- \frac{1}{2}}\\&\le {C_{\star }}({\Vert {w}\Vert } + {\Vert {w_x}\Vert }) {\Vert {v}\Vert } + C {\left| [{w}]\right| } {\Vert {v}\Vert _{\Gamma _{\!h}}} \\&\le {C_{\star }}\left( {\Vert {w}\Vert } + {\Vert {w_x}\Vert } + h^{-\frac{1}{2}}{\left| [{w}]\right| } \right) {\Vert {v}\Vert }. \end{aligned}$$

This completes the proof of Lemma 1. $\square$

##### Corollary 1

Under the same conditions as in Lemma 1, we have, for small enough *h*,

2.12 $$\begin{aligned} {{\mathcal {H}}} ((\partial _h^{\alpha } r) w,v) \le {C_{\star }}\left( {\Vert {w}\Vert } + {\Vert {w_x}\Vert } + h^{-\frac{1}{2}}{\left| [{w}]\right| }\right) {\Vert {v}\Vert }, \quad \forall \alpha \ge 0. \end{aligned}$$

##### Proof

The case $\alpha = 0$ has been proved in Lemma 1. For general $\alpha \ge 1$, let us start by using the relation (2.6e) for $\partial _h^{\alpha } r$ to obtain

$$\begin{aligned} { {{\mathcal {H}}} ((\partial _h^{\alpha } r) w,v) = {{\mathcal {H}}} ((\partial _{x}^{\alpha }r) w,v) + C h {{\mathcal {H}}} ( \Psi _{\!\alpha } w,v) } \end{aligned}$$

with *C* a positive constant and $\Psi _{\!\alpha }$ a smooth function. Next, applying (2.11a) in Lemma 1 to ${{\mathcal {H}}} ((\partial _{x}^{\alpha }r) w,v)$ and ${{\mathcal {H}}} ( \Psi _\alpha w,v)$, we have for small enough *h*

$$\begin{aligned} {{\mathcal {H}}} ((\partial _h^{\alpha } r) w,v)&\le {C_{\star }}(1 + Ch) \left( {\Vert {w}\Vert } + {\Vert {w_x}\Vert } + h^{-\frac{1}{2}}{\left| [{w}]\right| } \right) {\Vert {v}\Vert } \\&\le {C_{\star }}\left( {\Vert {w}\Vert } + {\Vert {w_x}\Vert } + h^{-\frac{1}{2}}{\left| [{w}]\right| }\right) {\Vert {v}\Vert }. \end{aligned}$$

This finishes the proof of Corollary 1. $\square$

##### Lemma 2

Suppose that *r*(*u*(*x*, *t*)) is smooth with respect to each variable. Then, for any $w \in H^{k+1}(\Omega _{h})$ and $v \in { V_{h}^\alpha }$, there holds

2.13 $$\begin{aligned} {{\mathcal {H}}} (r (w - P_h^- w),v) \le {C_{\star }}h^{k+1}{\Vert {v}\Vert }. \end{aligned}$$

##### Proof

Using the definition of the projection $P_h^-$ (2.9a), we have that $(w - P_h^- w)_{j'+\frac{1}{2}}^- = 0$, and thus

$$\begin{aligned} {{\mathcal {H}}} (r (w - P_h^- w),v) = ({r (w - P_h^- w)},{v_x}). \end{aligned}$$

Next, on each cell $I_{j'}$, we rewrite *r*(*u*(*x*, *t*)) as $r(u) = r(u_{j'}) + \left( r(u) - r(u_{j'}) \right)$ with $u_{j'} = {u(x_{j'},t)}$. Clearly, on each element $I_{j'}$, $| r(u) - r(u_{j'}) | \le {C_{\star }}h$ due to the smoothness of *r* and *u*. Using the orthogonality property of $P_h^-$ again (2.9b), we arrive at

$$\begin{aligned} {{\mathcal {H}}} (r (w - P_h^- w),v) = \left( ( r(u) - r(u_{j'}))(w - P_h^- w),v_x\right) \le {C_{\star }}h^{k+1}{\Vert {v}\Vert }, \end{aligned}$$

where we have used Cauchy–Schwarz inequality, inverse inequality (i) and the approximation property (2.10a) consecutively. $\square$

##### Corollary 2

Suppose that *r*(*u*(*x*, *t*)) is smooth with respect to each variable. Then, for any $w \in H^{k+1}(\Omega _{h})$, $v \in { V_{h}^\alpha }$, there holds

2.14 $$\begin{aligned} {{\mathcal {H}}} (\partial _h^{\alpha } (r (w - P_h^- w)),v) \le {C_{\star }}h^{k+1}{\Vert {v}\Vert },\quad \forall \alpha \ge 0. \end{aligned}$$

##### Proof

The case $\alpha = 0$ has been proved in Lemma 2. For $\alpha \ge 1$, by the Leibniz rule (2.6b) and taking into account the fact that both the divided difference operator $\partial _h$ and the projection operator $P_h^-$ are linear, we rewrite $\partial _h^{\alpha } (r (w - P_h^- w))$ as

$$\begin{aligned} \partial _h^{\alpha } (r (w - P_h^- w))&= {\sum _{\ell = 0}^\alpha }{\left( {\begin{array}{c}\alpha \\ \ell \end{array}}\right) }\partial _h^{\ell } r \left( x + \frac{\alpha - \ell }{2} h \right) \partial _h^{\alpha - \ell } (w-P_h^- w) \left( x - \frac{\ell }{2} h \right) \\&\triangleq {\sum _{\ell = 0}^\alpha }{\left( {\begin{array}{c}\alpha \\ \ell \end{array}}\right) }{\check{r}} \left( {\check{w}} - P_h^- {\check{w}} \right) \end{aligned}$$

with

$$\begin{aligned} {\check{r}} = \partial _h^{\ell } r \left( x + \frac{\alpha - \ell }{2} h \right) ,\quad {\check{w}} = \partial _h^{\alpha - \ell } w \left( x - \frac{\ell }{2} h \right) . \end{aligned}$$

Thus,

2.15 $$\begin{aligned} {{\mathcal {H}}} (\partial _h^{\alpha } (r (w - P_h^- w)),v) = {\sum _{\ell = 0}^\alpha }{\left( {\begin{array}{c}\alpha \\ \ell \end{array}}\right) } {{\mathcal {H}}} ({\check{r}} \left( {\check{w}} - P_h^- {\check{w}} \right) ,v). \end{aligned}$$

Clearly, by (2.6e), ${\check{r}}$ is also a smooth function with respect to each variable with leading term $\partial _{x}^{\ell }r \left( x + \frac{\alpha - \ell }{2} h \right)$. To complete the proof, we need only apply the same procedure as that in the proof of Lemma 2 to each ${{\mathcal {H}}}$ term on the right side of (2.15). $\square$

#### Regularity for the variable coefficient hyperbolic equations

Since the dual problem for the nonlinear hyperbolic equation is a variable coefficient equation, we need to recall a regularity result.

##### Lemma 3

[16] Consider the variable coefficient hyperbolic equation with a periodic boundary condition for all $t \in [0, T]$

2.16a $$\begin{aligned} \varphi _t(x,t) + a(x,t) \varphi _x(x,t)&= 0, \end{aligned}$$

2.16b $$\begin{aligned} \varphi (x,0)&= \varphi _0(x), \end{aligned}$$

where *a*(*x*, *t*) is a given smooth periodic function. For any $\ell \ge 0$, fix time *t* and $a(x,t) \in L^\infty ([0,T]; W^{2\ell +1,\infty }( {\Omega } ))$, then the solution of (2.16) satisfies the following regularity property

$$\begin{aligned} {\Vert {\varphi (x,t)}\Vert }_\ell \le C {\Vert {\varphi (x,0)}\Vert }_\ell , \end{aligned}$$

where *C* is a constant depending on ${\Vert {a}\Vert }_{L^\infty ([0,T];W^{2\ell +1,\infty }( {\Omega } ))}$.

#### SIAC filters

The SIAC filters are used to extract the hidden accuracy of DG methods, by means of a post-processing technique, which enhances the accuracy and reduces oscillations of the DG errors. The post-processing is a convolution with a kernel function $K_h^{\nu ,k+1}$ that is of compact support and is a linear combination of B-splines, scaled by the uniform mesh size,

$$\begin{aligned} K_h^{\nu ,k+1}(x) = \frac{1}{h} \sum _{\gamma \in {\mathbb {Z}}} c_\gamma ^{\nu , k+1} \psi ^{(k+1)}\left( \frac{x}{h} - \gamma \right) , \end{aligned}$$

where $\psi ^{(k+1)}$ is the B-spline of order $k+1$ obtained by convolving the characteristic function $\psi ^{(1)} = \chi$ of the interval $(-1/2,1/2)$ with itself *k* times. Additionally, the kernel function $K_h^{\nu ,k+1}$ should reproduce polynomials of degree $\nu - 1$ by convolution, which is used to determine the weights $c_\gamma ^{\nu , k+1}$. For more details, see [11].

The post-processing theory of SIAC filters is given in the following theorem.

##### Theorem 1

(Bramble and Schatz [5]) For $0< T < T^\star$, where $T^\star$ is the maximal time of existence of the smooth solution, let $u \in L^\infty ([0,T];H^{\nu }(\Omega ))$ be the exact solution of (1.1). Let $\Omega _0 + 2 \mathrm{{supp}} (K_h^{\nu ,k+1}(x)) \Subset \Omega$ and *U* be any approximation to *u*, then

$$\begin{aligned} {\Vert {u - K_h^{\nu ,k+1}\star U }\Vert }_{\Omega _0} \le \frac{h^\nu }{\nu !}C_1|u|_{\nu } + C_1 C_2 \sum _{\alpha \le k+1} \Vert {\partial _h^{\alpha } (u - U)}\Vert _{-{(k+1)},\Omega }, \end{aligned}$$

where $C_1$ and $C_2$ depend on $\Omega _0, k$, but is independent of *h*.

## $L^2$ norm error estimates for divided differences

By the post-processing theory [5, 11] (also see Theorem 1), it is essential to derive negative-order norm error estimates for divided differences, which depend heavily on their $L^2$ norm estimates. However, for both variable coefficient equations and nonlinear equations, it is highly nontrivial to derive $L^2$ norm error estimates for divided differences, and the technique used to prove convergence results for the DG error itself needs to be significantly changed.

### The main results in $L^2$ norm

Let us begin by denoting $e = u - u_h$ to be the error between the exact solution and numerical solution. Next, we split it into two parts; one is the projection error, denoted by ${\eta }= u - {{{\mathbb {Q}}}_h} u$, and the other is the projection of the error, denoted by $\xi = {{{\mathbb {Q}}}_h} u - u_h:= {{{\mathbb {Q}}}_h} e \in { V_{h}^\alpha }$. Here the projection ${{{\mathbb {Q}}}_h}$ is defined at each time level *t* corresponding to the sign variation of $f'(u)$; specifically, for any $t \in [0, T]$ and $x \in \Omega$, if $f'(u(x,t))>0$ we choose ${{{\mathbb {Q}}}_h} = P_h^-$, and if $f'(u(x,t))<0$, we take ${{{\mathbb {Q}}}_h} = P_h^+$.

We are now ready to state the main theorem for the $L^2$ norm error estimates.

#### Theorem 2

For any $0 \le \alpha \le k+1$, let $\partial _h^{\alpha } u$ be the exact solution of Eq. (2.1), which is assumed to be sufficiently smooth with bounded derivatives, and assume that $|f'(u)|$ is uniformly lower bounded by a positive constant. Let $\partial _h^{\alpha } u_h$ be the numerical solution of scheme (2.2) with initial condition $\partial _h^{\alpha } u_h(0) = {{{\mathbb {Q}}}_h} (\partial _h^{\alpha } u_0)$ when the upwind flux is used. For a uniform mesh of $\Omega = (a,b)$, if the finite element space ${ V_{h}^\alpha }$ of piecewise polynomials with arbitrary degree $k \ge 1$ is used, then for small enough *h* and any $T > 0$ there holds the following error estimate3.1where the positive constant ${C_{\star }}$ depends on the *u*, $\delta$, *T* and *f*, but is independent of *h*.

#### Corollary 3

Under the same conditions as in Theorem 2, if in addition $\alpha \ge 1$ we have the following error estimates:

3.2 $$\begin{aligned} {\Vert {\partial _h^{\alpha } (u - u_h)(T)}\Vert } \le {C_{\star }}h^{k + \frac{3}{2} - \frac{\alpha }{2}}. \end{aligned}$$

#### Proof

As shown in Corollary 2, we have that $\partial _h^{\alpha } {\eta }= \partial _h^{\alpha } u - P_h^-(\partial _h^{\alpha } u)$, and thus

3.3 $$\begin{aligned} {\Vert {\partial _h^{\alpha } {\eta }}\Vert } \le C h^{k+1}\Vert {\partial _h^{\alpha } u}\Vert _{{k+1}} \end{aligned}$$

by the approximation error estimate (2.10a). Now, the error estimate (3.2) follows by combining the triangle inequality and (3.1). $\square$

#### Remark 1

Clearly, the $L^2$ error estimates for the divided differences in Theorem 2 and Corollary 3 also hold for the variable coefficient equation (2.1) with $f(u) = a(x) u$ and $|a(x)| \ge \delta > 0$. In fact, for variable coefficient equations, we can obtain optimal $({k+1})$th order in the $L^2$ norm and thus $({2k+1})$th order in the negative-order norm; see Sect. 3.3.

#### Remark 2

The result with $\alpha = 0$ in Theorem 2 is indeed a superconvergence result towards a particular projection of the exact solution (supercloseness) that has been established in [18], which is a starting point for proving ${\Vert {\partial _h^{\alpha } \xi }\Vert }$ with $\alpha \ge 1$. For completeness, we list the superconvergence result for $\xi$ (zeroth order divided difference) as follows

3.4a $$\begin{aligned} {\Vert {\xi }\Vert ^2}+ {\int _{0}^T{{\vert \![{\xi }]\!\vert }^2}dt }&\le {C_{\star }}h^{2k+3}, \end{aligned}$$

3.4b $$\begin{aligned} {\Vert {\xi _x}\Vert } \le C h^{-1} {\Vert {{\mathbb {S}}}\Vert }&\le {C_{\star }}({\Vert {\xi _t}\Vert } + h^{k+1}),\end{aligned}$$

3.4c $$\begin{aligned} {\Vert {\xi _t}\Vert ^2} + {\int _{0}^T{{\vert \![{\xi _t}]\!\vert }^2}dt }&\le {C_{\star }}h^{2k+2}, \end{aligned}$$

where, on each element $I_j$, we have used $\xi = r_j + {\mathbb {S}}(x)(x-x_j)/h_j$ with $r_j = \xi (x_j)$ being a constant and ${\mathbb {S}}(x) \in P^{k-1}(I_j)$. Note that the proof of such superconvergence results requires that $|f'(u)|$ is uniformly lower bounded by a positive constant; for more details, see [18].

In the proof of Theorem 2, we have also obtained a generalized version about the $L^2$ norm estimates of $\xi$ in terms of the divided differences, their time derivatives, and spatial derivatives. To simplify notation, for an arbitrary multi-index $\beta = (\beta _1,\beta _2)$, we denote by $\partial _{\mathfrak M}^{\beta } (\cdot )$ the mixed operator containing divided differences and time derivatives of a given function, namely

3.5 $$\begin{aligned} \partial _{\mathfrak M}^{\beta } (\cdot ) = \partial _h^{\beta _1} \partial _{t}^{\beta _2}(\cdot ) . \end{aligned}$$

#### Corollary 4

Under the same conditions as in Theorem 2, for $\beta _0 = 0, 1$ and a multi-index $\beta = (\beta _1,\beta _2)$ with $|\beta | = \beta _1 + \beta _2 \le {k+1}$, we have the following unified error estimate:

$$\begin{aligned} {\Vert {\partial _{x}^{\beta _0}\partial _{\mathfrak M}^{\beta } \xi (T)}\Vert } \le {C_{\star }}h^{k+\frac{3}{2} -\frac{|\beta '|}{2}}, \end{aligned}$$

where $$|\beta '| = \beta _0 + |\beta |$$.

### Proof of the main results in the $L^2$ norm

Similar to the discussion of the DG discretization operator properties in Sect. 2.2.4, without loss of generality, we will only consider the case $f'(u(x,t)) \ge \delta > 0$ for all $(x,t) \in \Omega \times [0,T]$; the case of $f'(u(x,t)) \le - \delta < 0$ is analogous. Therefore, we take the upwind numerical flux as $\hat{f} = f(u_h^-)$ on each cell interface and choose the projection as ${{{\mathbb {Q}}}_h} = P_h^-$ on each cell, and the initial condition is chosen as $\partial _h^{\alpha } u_h(0) = P_h^- (\partial _h^{\alpha } u_0)$. Since the case $\alpha = 0$ has already been proven in [18] (see (3.4a)), we need only to consider $1 \le \alpha \le k+1$. In order to clearly display the main ideas of how to perform the $L^2$ norm error estimates for divided differences, in the following two sections we present the detailed proof for Theorem 2 with $\alpha = 1$ and $\alpha = 2$, respectively; the general cases with $3 \le \alpha \le k+1$ $(k \ge 2)$ can be proven by induction, which are omitted to save space.

#### Analysis for the first order divided difference

For $\alpha = 1$, the DG scheme (2.2) becomes

$$\begin{aligned} \left( {(\partial _{h} {u_h})}_t,v_h\right) _{j'} = {{\mathcal {H}}}_{j'}\left( \partial _{h} {f(u_h)},v_h\right) \end{aligned}$$

with $j' = j+\frac{1}{2}$, which holds for any $v_h \in { V_{h}^\alpha }$ and $j = 1, \ldots , N$. By Galerkin orthogonality and summing over all $j'$, we have the error equation

3.6 $$\begin{aligned} \left( \partial _{h} {e_t},v_h\right) = {{\mathcal {H}}} (\partial _{h} {(f(u) - f(u_h))},v_h) \end{aligned}$$

for all $v_h \in { V_{h}^\alpha } .$ To simplify notation, we would like to denote $\partial _{h} {e} := \bar{e}= {\bar{\eta }}+ \bar{\xi }$ with ${\bar{\eta }}= \partial _{h} {{\eta }}, \bar{\xi }= \partial _{h} {\xi }$. If we now take $v_h = \bar{\xi }$, we get the following identity

3.7 $$\begin{aligned} \frac{1}{2}\frac{d}{dt}{\Vert {\bar{\xi }}\Vert ^2}+ \left( {\bar{\eta }}_t,\bar{\xi }\right) = {{\mathcal {H}}} (\partial _{h} {(f(u) - f(u_h))},\bar{\xi }). \end{aligned}$$

The estimate for the right side of (3.7) is complicated, since it contains some integral terms involving mixed order divided differences of $\xi$, namely $\xi$ and $\bar{\xi }$, which is given in the following lemma.

##### Lemma 4

Suppose that the conditions in Theorem 2 hold. Then we have3.8where the positive constants *C* and ${C_{\star }}$ are independent of *h* and $u_h.$

##### Proof

Let us start by using the second order Taylor expansion with respect to the variable *u* to write out the nonlinear terms, namely $f(u) - f(u_h)$ and $f(u) - f(u_h^-)$, as

3.9a $$\begin{aligned} f(u) - f(u_h)&= f'(u) \xi + f'(u) {\eta }- R_1 e^2, \end{aligned}$$

3.9b $$\begin{aligned} f(u) - f(u_h^-)&= f'(u) \xi ^- + f'(u) {\eta }^- - R_2 (e^-)^2, \end{aligned}$$

where $R_1 = \int _0^1 (1 - \mu ) f''(u + \mu (u_h - u)) d\mu$ and $R_2 = \int _0^1 (1 - \nu ) f''(u + \nu (u_h^- - u)) d\nu$ are the integral form of the remainders of the second order Taylor expansion. We would like to emphasize that the various order spatial derivatives, time derivatives and divided differences of $R_1$ are all bounded uniformly due to the smoothness of *f* and *u*. Thus,

$$\begin{aligned} {{\mathcal {H}}} (\partial _{h} {(f(u) - f(u_h))},\bar{\xi })&= {{\mathcal {H}}} (\partial _{h} {(f'(u) \xi )},\bar{\xi }) + {{\mathcal {H}}} (\partial _{h} {(f'(u) {\eta })},\bar{\xi }) - {{\mathcal {H}}} (\partial _{h} {(R_1 e^2)},\bar{\xi }) \\&\triangleq {\mathcal {J}} + {\mathcal {K}} - {\mathcal {L}}, \end{aligned}$$

which will be estimated separately below.

To estimate ${\mathcal {J}}$, we employ the Leibniz rule (2.6b), and rewrite $\partial _{h} {(f'(u) \xi })$ as

$$\begin{aligned} \partial _{h} {(f'(u) \xi )} = f'(u(x+h/2)) \bar{\xi }(x) + (\partial _{h} {f'(u(x))}) \xi (x - h/2), \end{aligned}$$

and thus,

$$\begin{aligned} {\mathcal {J}} = {{\mathcal {H}}} (f'(u) \bar{\xi },\bar{\xi }) + {{\mathcal {H}}} ( (\partial _{h} {f'(u)}) \xi ,\bar{\xi }) \triangleq {\mathcal {J}}_1 + {\mathcal {J}}_2, \end{aligned}$$

where we have omitted the dependence of *x* for convenience if there is no confusion, since the proof of (2.11b) is still valid even if $f'(u)$ and $\bar{\xi }$ are evaluated at different *x*; see proof of (2.11b) in Sect. 2.2.4. A direct application of Lemma 1 together with the assumption that $f'(u) \ge \delta >0$, (2.11b), leads to the estimate for ${\mathcal {J}}_1$: 3.10aBy Corollary 1, we arrive at the estimate for ${\mathcal {J}}_2$:

3.10b $$\begin{aligned} {\mathcal {J}}_2 \le {C_{\star }}\left( {\Vert {\xi }\Vert }+ {\Vert {\xi _x}\Vert } + h^{-\frac{1}{2}} {\left| [{\xi }]\right| }\right) {\Vert {\bar{\xi }}\Vert }. \end{aligned}$$

Substituting (3.4a)–(3.4c) into (3.10b), and combining with (3.10a), we have, after a straightforward application of Young’s inequality, that3.11Let us now move on to the estimate of ${\mathcal {K}}$. By Corollary 2, we have

3.12 $$\begin{aligned} {\mathcal {K}} \le {C_{\star }}h^{k+1}{\Vert {\bar{\xi }}\Vert }. \end{aligned}$$

To estimate ${\mathcal {L}}$, let us first employ the identity (2.6b) and rewrite $\partial _{h} {(R_1 e^2)}$ as

$$\begin{aligned} \partial _{h} {(R_1 e^2)}&= R_1(u(x+h/2)) \partial _{h} {e^2} + \partial _{h} {R_1(u(x))} e^2(x - h/2) \\&= R_1(u(x+h/2)) \bar{e}(x) (e(x+h/2) + e(x - h/2))\\&\quad + \partial _{h} {R_1(u(x))} e^2(x - h/2)\\&\triangleq D_1 + D_2. \end{aligned}$$

Consequently,

$$\begin{aligned} {\mathcal {L}} = {{\mathcal {H}}} (D_1,\bar{\xi }) + {{\mathcal {H}}} (D_2,\bar{\xi }). \end{aligned}$$

It is easy to show, for the high order nonlinear term ${{\mathcal {H}}} (D_1,\bar{\xi })$, that

3.13 $$\begin{aligned} {{\mathcal {H}}} (D_1,\bar{\xi })&\le {C_{\star }}{\Vert {e}\Vert _{\infty }} \left( {\Vert {\bar{e}}\Vert }{\Vert {\bar{\xi }_x}\Vert } + {\Vert {\bar{e}}\Vert _{\Gamma _{\!h}}}{\Vert {\bar{\xi }}\Vert _{\Gamma _{\!h}}}\right) \nonumber \\&\le {C_{\star }}h^{-1} {\Vert {e}\Vert _{\infty }} \left( {\Vert {\bar{\xi }}\Vert }+ {\Vert {{\bar{\eta }}}\Vert }+ h^\frac{1}{2}{\Vert {{\bar{\eta }}}\Vert _{\Gamma _{\!h}}}\right) {\Vert {\bar{\xi }}\Vert }\nonumber \\&\le {C_{\star }}h^{-1} {\Vert {e}\Vert _{\infty }} \left( {\Vert {\bar{\xi }}\Vert }+ h^{k+1}\right) {\Vert {\bar{\xi }}\Vert }, \end{aligned}$$

where in the first step we have used the Cauchy–Schwarz inequality, in the second step we have used the inverse properties (i) and (ii), and in the last step we have employed the interpolation properties (3.3). We see that in order to deal with the nonlinearity of *f* we still need to have a bound for ${\Vert {e}\Vert _{\infty }}$. Due to the superconvergence result (3.4a), we conclude, by combining inverse inequality (iii) and the approximation property (2.10b), that

3.14 $$\begin{aligned} {\Vert {e}\Vert _{\infty }} \le C h^{k+1}. \end{aligned}$$

Therefore, for small enough *h*, we have

3.15a $$\begin{aligned} {{\mathcal {H}}} (D_1,\bar{\xi }) \le {C_{\star }}{\Vert {\bar{\xi }}\Vert ^2}+ {C_{\star }}h^{k+1}{\Vert {\bar{\xi }}\Vert }. \end{aligned}$$

By using analysis similar to that in the proof of (3.13), we have, for ${{\mathcal {H}}} (D_2,\bar{\xi })$, that

$$\begin{aligned} {{\mathcal {H}}} (D_2,\bar{\xi }) \le {C_{\star }}h^{-1} {\Vert {e}\Vert _{\infty }} \left( {\Vert {\xi }\Vert }+ h^{k+1}\right) {\Vert {\bar{\xi }}\Vert }. \end{aligned}$$

As a consequence, by (3.14) and (3.4a)

3.15b $$\begin{aligned} {{\mathcal {H}}} (D_2,\bar{\xi }) \le {C_{\star }}h^{k+1}{\Vert {\bar{\xi }}\Vert }. \end{aligned}$$

A combination of (3.15a) and (3.15b) produces a bound for ${\mathcal {L}}$:

3.16 $$\begin{aligned} {\mathcal {L}} \le {C_{\star }}{\Vert {\bar{\xi }}\Vert ^2}+ {C_{\star }}h^{k+1}{\Vert {\bar{\xi }}\Vert }. \end{aligned}$$

To complete the proof of Lemma 4, we need only combine (3.11), (3.12), (3.16) and use Young’s inequality. $\square$

We are now ready to derive the $L^2$ norm estimate for $\bar{\xi }$. To do this, let us begin by inserting the estimate (3.8) into (3.7) and taking into account the bound for ${\bar{\eta }}$ in (3.3) and thus ${\bar{\eta }}_t$ to get, after an application of Cauchy–Schwarz inequality and Young’s inequality, thatNext, we integrate the above inequality with respect to time between 0 and *T* and note the fact that $\bar{\xi }(0) = 0$ due to $\xi (0) = 0$ to obtainwhere we have used the superconvergence result (3.4a). An application of Gronwall’s inequality leads to the desired result3.17This finishes the proof of Theorem 2 for $\alpha = 1$.

##### Remark 3

We can see that the estimates (3.17) for the $L^2$ norm and the jump seminorm of $\bar{\xi }$ are based on the corresponding results for $\xi$ in Remark 2, which are half an order lower than that of $\xi$. This is mainly due to the hybrid of different order divided differences of $\xi$, namely $\xi$ and $\bar{\xi }$, and thus the application of inverse property (ii). It is natural that the proof for the high order divided difference of $\xi$, say $\partial _h^{2} \xi$, should be based on the corresponding lower order divided difference results of $\xi$ ($\xi$ and $\bar{\xi }$) that have already been established; see Sect. 3.2.2 below.

#### Analysis for the second order divided difference

For $\alpha = 2$, the DG scheme (2.2) becomes

$$\begin{aligned} \left( {(\partial _{h}^{2} {u_h})}_t,v_h\right) _{j'} = {{\mathcal {H}}}_{j'}\left( \partial _{h}^{2} {f(u_h)},v_h\right) \end{aligned}$$

with $j' = j$, which holds for any $v_h \in { V_{h}^\alpha }$ and $j = 1, \ldots , N$. By Galerkin orthogonality and summing over all *j*, we have the error equation

3.18 $$\begin{aligned} \left( \partial _{h}^{2} {e_t},v_h\right) = {{\mathcal {H}}} (\partial _{h}^{2} {(f(u) - f(u_h))},v_h) \end{aligned}$$

for all $v_h \in { V_{h}^\alpha } .$ To simplify notation, we would like to denote $\partial _{h}^{2} {e} := \tilde{e}= {\tilde{\eta }}+ \tilde{\xi }$ with ${\tilde{\eta }}= \partial _{h}^{2} {{\eta }}, \tilde{\xi }= \partial _{h}^{2} {\xi }$. If we now take $v_h = \tilde{\xi }$, we get the following identity

3.19 $$\begin{aligned} \frac{1}{2}\frac{d}{dt}{\Vert {\tilde{\xi }}\Vert ^2}+ \left( {\tilde{\eta }}_t,\tilde{\xi }\right) = {{\mathcal {H}}} (\partial _{h}^{2} {(f(u) - f(u_h))},\tilde{\xi }). \end{aligned}$$

The estimate for right side of (3.19) is rather complicated, since it contains some integral terms involving mixed order divided differences of $\xi$, namely $\xi$, $\bar{\xi }$ and $\tilde{\xi }$, which is given in the following Proposition.

##### Proposition 1

Suppose that the conditions in Theorem 2 hold. Then we have3.20where the positive constants *C* and ${C_{\star }}$ are independent of *h* and $u_h.$

##### Proof

By the second order Taylor expansion (3.9), we have

3.21 $$\begin{aligned} {{\mathcal {H}}} (\partial _{h}^{2} {(f(u) - f(u_h))},\tilde{\xi })&= {{\mathcal {H}}} (\partial _{h}^{2} {(f'(u) \xi )},\tilde{\xi }) + {{\mathcal {H}}} (\partial _{h}^{2} {(f'(u) {\eta })},\tilde{\xi }) \nonumber \\&\quad - {{\mathcal {H}}} (\partial _{h}^{2} {(R_1 e^2)},\tilde{\xi }) \nonumber \\&\triangleq {\mathcal {P}} + {\mathcal {Q}} - {\mathcal {S}}, \end{aligned}$$

which will be estimated one by one below.

To estimate ${\mathcal {P}}$, we use the Leibniz rule (2.6b), to rewrite $\partial _{h}^{2} {(f'(u) \xi )}$ as

$$\begin{aligned} \partial _{h}^{2} {(f'(u) \xi )}= & {} f'(u(x+h)) \tilde{\xi }(x) + 2 \partial _{h} {f'(u(x+h/2))} \bar{\xi }(x - h/2)\\&+ \,\partial _{h}^{2} {f'(u(x))} \xi (x-h), \end{aligned}$$

and thus,

$$\begin{aligned} {\mathcal {P}} = {{\mathcal {H}}} (f'(u) \tilde{\xi },\tilde{\xi }) + 2 {{\mathcal {H}}} ((\partial _{h} {f'(u)}) \bar{\xi },\tilde{\xi }) + {{\mathcal {H}}} ((\partial _{h}^{2} {f'(u)}) \xi ,\tilde{\xi }) \triangleq {\mathcal {P}}_1 + {\mathcal {P}}_2 + {\mathcal {P}}_3, \end{aligned}$$

where we have omitted the dependence of *x* for convenience if there is no confusion. A direct application of Lemma 1 together with the assumption that $f'(u) \ge \delta >0$, (2.11b), produces the estimate for ${\mathcal {P}}_1$: 3.22aBy Corollary 1, we arrive at the estimates for ${\mathcal {P}}_2$ and ${\mathcal {P}}_3$:

3.22b $$\begin{aligned} {\mathcal {P}}_2&\le {C_{\star }}\left( {\Vert {\bar{\xi }}\Vert }+ {\Vert {\bar{\xi }_x}\Vert } + h^{-\frac{1}{2}} {\left| [{\bar{\xi }}]\right| }\right) {\Vert {\tilde{\xi }}\Vert },\end{aligned}$$

3.22c $$\begin{aligned} {\mathcal {P}}_3&\le {C_{\star }}\left( {\Vert {\xi }\Vert }+ {\Vert {\xi _x}\Vert } + h^{-\frac{1}{2}} {\left| [{\xi }]\right| }\right) {\Vert {\tilde{\xi }}\Vert }. \end{aligned}$$

Substituting (3.4a)–(3.4c), (3.17) into (3.22b), (3.22c), and combining with (3.22a), we have, after a straightforward application of Young’s inequality, that3.23For terms on the right side of (3.23), although we have information about  and  as shown in (3.4a) and (3.17), we still need a suitable bound for ${\Vert {{\bar{\xi }}_x}\Vert }$, which is given in the following lemma.

##### Lemma 5

Suppose that the conditions in Theorem 2 hold. Then we have

3.24 $$\begin{aligned} {\Vert {{\bar{\xi }}_x}\Vert }\le {C_{\star }}( {\Vert {{\bar{\xi }}_t}\Vert }+ h^{k+1}), \end{aligned}$$

where ${C_{\star }}$ depends on *u* and $\delta$ but is independent of *h* and $u_h$.

The proof of this lemma is given in the appendix. Up to now, we see that we still need to have a bound for ${\Vert {{\bar{\xi }}_t}\Vert }$. In fact, the proof for ${\Vert {{\bar{\xi }}_t}\Vert }$ would require additional bounds for ${\Vert {({\xi }_t)_x}\Vert }$ and ${\Vert {\xi _{tt}}\Vert }$, whose results are shown in Lemmas 6 and 7.

##### Lemma 6

Suppose that the conditions in Theorem 2 hold. Then we have

3.25 $$\begin{aligned} {\Vert {({\xi }_t)_x}\Vert }\le {C_{\star }}( {\Vert {\xi _{tt}}\Vert }+ h^{k+1}). \end{aligned}$$

The proof of Lemma 6 follows along a similar argument as that in the proof of Lemma 5, so we omit the details here.

##### Lemma 7

Suppose that the conditions in Theorem 2 hold. Then we have3.26


The proof of this lemma is deferred to the appendix. Based on the above two lemmas, we are able to prove the bound for ${\Vert {{\bar{\xi }}_t}\Vert }$ in Lemma 8, whose proof is deferred to the appendix.

##### Lemma 8

Suppose that the conditions in Theorem 2 hold. Then we have3.27where ${C_{\star }}$ depends on *u* and $\delta$ but is independent of *h* and $u_h$.

We now collect the estimates in Lemmas 5 and 8 into (3.23) to get3.28Let us now move on to the estimate of ${\mathcal {Q}}$. By Corollary 2, we have

3.29 $$\begin{aligned} {\mathcal {Q}} \le {C_{\star }}h^{k+1}{\Vert {\tilde{\xi }}\Vert }. \end{aligned}$$

To estimate ${\mathcal {S}}$, let us first employ the identity (2.6b) and rewrite $\partial _{h}^{2} {(R_1 e^2)}$ as

$$\begin{aligned} \partial _{h}^{2} {(R_1 e^2)}&= R_1(u(x+h)) \partial _{h}^{2} {e^2} + 2 \partial _{h} {R_1(u(x+h/2))} \partial _{h} {e^2}(x - h/2) \\&\quad + \partial _{h}^{2} {R_1(u(x))} e^2(x-h )\\&\triangleq E_1 + E_2 + E_3, \end{aligned}$$

where

$$\begin{aligned} E_1&= R_1(u(x+h)) \left( e(x+h) \tilde{e}(x) + 2 \bar{e}(x+h/2)\bar{e}(x-h/2) + \tilde{e}(x) e(x-h)\right) , \\ E_2&= 2 \partial _{h} {R_1(u(x+h/2))}\bar{e}(x-h/2) \left( e(x) + e(x-h)\right) , \\ E_3&= \partial _{h}^{2} {R_1(u(x))} e^2(x-h ). \end{aligned}$$

Thus,

$$\begin{aligned} {\mathcal {S}} = {{\mathcal {H}}} (E_1,\tilde{\xi }) + {{\mathcal {H}}} (E_2,\tilde{\xi }) + {{\mathcal {H}}} (E_3,\tilde{\xi }) \triangleq {\mathcal {S}}_1 + {\mathcal {S}}_2 +{\mathcal {S}}_3. \end{aligned}$$

By using analysis similar to that in the proof of (3.13), we get

$$\begin{aligned} {\mathcal {S}}_1&\le {C_{\star }}h^{-1} ({\Vert {e}\Vert _{\infty }} + {\Vert {\bar{e}}\Vert _{\infty }})\left( {\Vert {\tilde{\xi }}\Vert }+ {\Vert {\bar{\xi }}\Vert }+ h^{k+1}\right) {\Vert {\tilde{\xi }}\Vert } \\&\le C \left( {\Vert {\tilde{\xi }}\Vert }+ {\Vert {\bar{\xi }}\Vert }+ h^{k+1}\right) {\Vert {\tilde{\xi }}\Vert },\\ {\mathcal {S}}_2&\le {C_{\star }}h^{-1} {\Vert {e}\Vert _{\infty }} \left( {\Vert {\bar{\xi }}\Vert }+ h^{k+1}\right) {\Vert {\tilde{\xi }}\Vert } \le C \left( {\Vert {\bar{\xi }}\Vert }+ h^{k+1}\right) {\Vert {\tilde{\xi }}\Vert },\\ {\mathcal {S}}_3&\le {C_{\star }}h^{-1} {\Vert {e}\Vert _{\infty }} \left( {\Vert {\xi }\Vert }+ h^{k+1}\right) {\Vert {\tilde{\xi }}\Vert } \le C \left( {\Vert {\xi }\Vert }+ h^{k+1}\right) {\Vert {\tilde{\xi }}\Vert }, \end{aligned}$$

where we have used the fact that for $k \ge 1$ and small enough *h*, ${C_{\star }}h^{-1} ({\Vert {e}\Vert _{\infty }} + {\Vert {\bar{e}}\Vert _{\infty }}) \le C$; for more details, see the appendix. Consequently

3.30 $$\begin{aligned} {\mathcal {S}} \le C \left( {\Vert {\tilde{\xi }}\Vert }+ {\Vert {\bar{\xi }}\Vert }+ {\Vert {\xi }\Vert }+ h^{k+1}\right) {\Vert {\tilde{\xi }}\Vert }. \end{aligned}$$

Collecting the estimates (3.28)–(3.30) into (3.21) and taking into account (3.4a) and (3.17), we getThis finishes the proof of Proposition 1. $\square$

We are now ready to derive the $L^2$ norm estimate for $\tilde{\xi }$. To do this, we begin by combining (3.19) and (3.20) to getNext, integrate the above inequality with respect to time between 0 and *T* and use $\xi (0) = 0$ (and thus $\tilde{\xi }(0) = \partial _{h}^{2} {\xi }(0) = 0$) to obtainby the estimates (3.4a) and (3.17). An application of Gronwall’s inequality leads to the desired result3.31This completes the proof of Theorem 2 with $\alpha = 2$.

##### Remark 4

Through the proof of Theorem 2 with $\alpha = 2$, ${\Vert {\tilde{\xi }}\Vert }$, we can see that apart from the bounds for ${\Vert {\xi }\Vert }, {\Vert {\xi _x}\Vert }, {\Vert {\xi _t}\Vert }$ that have already been obtained for proving ${\Vert {\bar{\xi }}\Vert }$, we require additional bounds for ${\Vert {{\bar{\xi }}_x}\Vert }, {\Vert {{\bar{\xi }}_t}\Vert }, {\Vert {({\xi }_t)_x}\Vert },$ and ${\Vert {\xi _{tt}}\Vert }$, as shown in Lemmas 5–8. The proof for the $L^2$ norm estimates for higher order divided differences are more technical and complicated, and it would require bounds regarding lower order divided differences as well as its corresponding spatial and time derivatives. For example, when $\alpha = 3$, in addition to the abounds aforementioned, we need to establish the bounds for ${\Vert {{\tilde{\xi }}_x}\Vert }, {\Vert {{\tilde{\xi }}_t}\Vert }, {\Vert {({{\bar{\xi }}_t)}_x}\Vert }, {\Vert {{\bar{\xi }}_{tt}}\Vert },{\Vert {(\xi _{tt})_x}\Vert }$ and ${\Vert {\xi _{ttt}}\Vert }$. Thus, Theorem 2 can be proven along the same lines for general $\alpha \le {k+1}$. Finally, we would like to point out that the corresponding results on the jump seminorm for various order divided differences and time derivatives of $\xi$ are useful, which play an important role in deriving Theorem 2.

### Variable coefficient case

#### The main results

In this section we consider the $L^2$ error estimates for divided differences for the variable coefficient equation (1.1) with $f(u) = a(x)u$. Similar to the nonlinear hyperbolic case, to obtain a suitable bound for the $L^2$ norm the numerical flux should be chosen as an upwind flux. Moreover, the analysis requires a condition that |*a*(*x*)| is uniformly lower bounded by a positive constant. Without loss of generality, we only consider $a(x)\ge \delta >0$, and thus the DG scheme is

3.32 $$\begin{aligned} \left( {(\partial _h^{\alpha } u_h)}_t,v_h\right) = {{\mathcal {H}}} (\partial _h^{\alpha } (a u_h),v_h) \end{aligned}$$

for $v_h \in { V_{h}^\alpha }$. We will use the same notation as before.

For nonlinear hyperbolic equations, the loss of order in Theorem 2 is mainly due to the lack of control for the interface jump terms arising from (2.11a) in the superconvergence relation, for example, (3.4b), (3.24) and (3.25). Fortunately, for variable coefficient hyperbolic equations, we can establish a stronger superconvergence relation between the spatial derivative as well as interface jumps of the various order divided difference of $\xi$ and its time derivatives; see (3.37b) below. Thus, optimal $L^2$ error estimates of order ${k+1}$ are obtained.

Prior to stating our main theorem, we would like to present convergence results for time derivatives of $\xi$, which is slightly different to those for nonlinear hyperbolic equations.

##### Lemma 9

Let *u* be the exact solution of the variable coefficient hyperbolic Eq. (1.1) with $f(u) = a(x) u$, which is assumed to be sufficiently smooth with bounded derivatives. Let ${u_h}$ be the numerical solution of scheme (3.32) $(\alpha = 0)$ with initial condition ${u_h(0)} = {{{\mathbb {Q}}}_h} {u_0},$ $( {{{\mathbb {Q}}}_h} = P^\pm _h )$ when the upwind flux is used. For regular triangulations of $\Omega = (a,b)$, if the finite element space ${ V_{h}^\alpha }$ of piecewise polynomials with arbitrary degree $k \ge 0$ is used, then for any $m \ge 0$ and any $T > 0$ there holds the following error estimate

3.33 $$\begin{aligned} {\Vert {\partial _{t}^{m}\xi (T)}\Vert } \le C h^{k+1}, \end{aligned}$$

where the positive constant *C* depends on *u*, *T* and *a*, but is independent of *h*.

The proof of this lemma is postponed to the appendix.

We are now ready to state our main theorem.

##### Theorem 3

For any $\alpha \ge 1$, let $\partial _h^{\alpha } u$ be the exact solution of the problem (2.1) with $f(u) = a(x) u$, which is assumed to be sufficiently smooth with bounded derivatives, and assume that |*a*(*x*)| is uniformly lower bounded by a positive constant. Let $\partial _h^{\alpha } u_h$ be the numerical solution of scheme (3.32) with initial condition $\partial _h^{\alpha } u_h(0) = {{{\mathbb {Q}}}_h} (\partial _h^{\alpha } u_0)$ when the upwind flux is used. For a uniform mesh of $\Omega = (a,b)$, if the finite element space ${ V_{h}^\alpha }$ of piecewise polynomials with arbitrary degree $k \ge 0$ is used, then for any $T > 0$ there holds the following error estimate

3.34 $$\begin{aligned} {\Vert {\partial _h^{\alpha } \xi (T)}\Vert } \le C h^{k+1}, \end{aligned}$$

where the positive constant *C* depends on *u*, $\delta$, *T* and *a*, but is independent of *h*.

##### Remark 5

Based on the optimal error estimates for ${\Vert {\partial _h^{\alpha } \xi }\Vert }$ together with approximation error estimates (3.3) and using the duality argument in [19], we can obtain the negative-order norm estimates

3.35 $$\begin{aligned} \Vert {\partial _h^{\alpha } (u - u_h)(T)}\Vert _{-{(k+1)},\Omega }\le C h^{2k+1}, \end{aligned}$$

and thus

3.36 $$\begin{aligned} {\Vert {u - K_h^{\nu ,k+1} \star u_h}\Vert } \le C h^{2k+1}. \end{aligned}$$

For more details, see [5, 19] and also Sect. 4 below.

#### Proof of main results

We shall prove Theorem 3 for general $\alpha \ge 1$. First we claim that if we can prove the following three inequalities

3.37a $$\begin{aligned} {\Vert {\partial _h^{m} \xi }\Vert }&\le C h^{k+1}, \quad \forall ~ 0\le m \le \alpha - 1,\end{aligned}$$

3.37b $$\begin{aligned} {\Vert {(\partial _{\mathfrak M}^{\beta } \xi )_x}\Vert } + h^{-\frac{1}{2}} {\left| [{\partial _{\mathfrak M}^{\beta } \xi }]\right| }&\le C \left( {\Vert {\partial _h^{\beta _1} \partial _{t}^{\beta _2+1}\xi }\Vert } + h^{k+1}\right) ,\quad \forall ~ |\beta | = \beta _1 + \beta _2 \le \alpha - 1,\end{aligned}$$

3.37c $$\begin{aligned} {\Vert {\partial _{\mathfrak M}^{\gamma } \xi }\Vert }&\le C h^{k+1},~~\forall ~ |\gamma | \le \alpha \quad and \quad \gamma \ne (\alpha ,0), \end{aligned}$$

where $\partial _{\mathfrak M}^{\beta } \xi = \partial _h^{\beta _1} \partial _{t}^{\beta _2}\xi$ represents the mixed operator containing divided differences and time derivatives of $\xi$ that has already been defined in (3.5), then ${\Vert {\partial _h^{\alpha } \xi }\Vert } \le C h^{k+1}$. In what follows, we sketch the verification of this claim. To do that, we start by taking $v_h = \partial _h^{\alpha } \xi$ in the following error equation

$$\begin{aligned} \left( \partial _h^{\alpha } e_t,v_h\right) = {{\mathcal {H}}} (\partial _h^{\alpha } (a\xi ),v_h) + {{\mathcal {H}}} (\partial _h^{\alpha } (a{\eta }),v_h), \end{aligned}$$

which is

3.38 $$\begin{aligned} \frac{1}{2}\frac{d}{dt}{\Vert {\partial _h^{\alpha } \xi }\Vert ^2} + \left( \partial _h^{\alpha } {\eta }_t,\partial _h^{\alpha } \xi \right) = {{\mathcal {H}}} (\partial _h^{\alpha } (a\xi ),\partial _h^{\alpha } \xi ) + {{\mathcal {H}}} (\partial _h^{\alpha } (a{\eta }),\partial _h^{\alpha } \xi ). \end{aligned}$$

Next, consider the term ${{\mathcal {H}}} (\partial _h^{\alpha } (a\xi ),\partial _h^{\alpha } \xi )$. Use Leibniz rule (2.6b) to rewrite $\partial _h^{\alpha } (a\xi )$ and employ (2.11a), (2.11b) in Lemma 1 to get the bound

$$\begin{aligned} {{\mathcal {H}}} (\partial _h^{\alpha } (a\xi ),\partial _h^{\alpha } \xi ) \le C {\Vert {\partial _h^{\alpha } \xi }\Vert ^2} + C h^{k+1}{\Vert {\partial _h^{\alpha } \xi }\Vert }, \end{aligned}$$

where we have also used the relations (3.37a)–(3.37c). For the estimate of ${{\mathcal {H}}} (\partial _h^{\alpha } (a{\eta }),\partial _h^{\alpha } \xi )$, we need only use Corollary 2 to get

$$\begin{aligned} {{\mathcal {H}}} (\partial _h^{\alpha } (a{\eta }),\partial _h^{\alpha } \xi ) \le C h^{k+1}{\Vert {\partial _h^{\alpha } \xi }\Vert }. \end{aligned}$$

Collecting above two estimates into (3.38) and using Cauchy–Schwarz inequality as well as Gronwall’s inequality, we finally get

$$\begin{aligned} {\Vert {\partial _h^{\alpha } \xi }\Vert } \le C h^{k+1}. \end{aligned}$$

The claim is thus verified.

In what follows, we will prove (3.37) by induction.

**Step 1**  For $\alpha = 1$, ${\Vert {\xi }\Vert } \le C h^{k+1}$ is well known, and thus (3.37a) is valid for $\alpha = 1$. Moreover, (3.37c), namely ${\Vert {\xi _t}\Vert } \le C h^{k+1}$ has been given in (3.4c); see [18]. To complete the proof for $\alpha = 1$, we need only to establish the following relation

3.39 $$\begin{aligned} {\Vert {\xi _x}\Vert } + h^{-\frac{1}{2}} {\left| [{\xi }]\right| } \le C \left( {\Vert {\xi _t}\Vert } + h^{k+1}\right) . \end{aligned}$$

##### Proof

Noting the relation (3.4b), we need only to prove

3.40 $$\begin{aligned} h^{-\frac{1}{2}} {\left| [{\xi }]\right| } \le C \left( {\Vert {\xi _t}\Vert } + h^{k+1}\right) . \end{aligned}$$

To do that, we consider the cell error equation

$$\begin{aligned} \left( e_t,v_h\right) _j = {{\mathcal {H}}}_{j}\left( ae,v_h\right) = {{\mathcal {H}}}_{j}\left( a \xi ,v_h\right) + {{\mathcal {H}}}_{j}\left( a {\eta },v_h\right) , \end{aligned}$$

which holds for any $v_h \in { V_{h}^\alpha }$ and $j = 1, \ldots , N$. If we now take $v_h = 1$ in the above identity and use the strong form (2.3b) for ${{\mathcal {H}}}_{j}\left( a \xi ,v_h\right)$, we get

$$\begin{aligned} \left( e_t,1\right) _j = - \left( (a\xi )_x,1\right) _j - (a[\![{\xi }]\!])_{j - \frac{1}{2}} + {{\mathcal {H}}}_{j}\left( a {\eta },1\right) \triangleq - W_1 - W_2 + W_3. \end{aligned}$$

It follows from the assumption $|a(x)| \ge \delta > 0$ that

3.41 $$\begin{aligned} \delta | {[\![{\xi }]\!]}_{j - \frac{1}{2}}| \le |W_2| \le |W_1| + |W_3| + |\left( e_t,1\right) _j|. \end{aligned}$$

By Cauchy–Schwarz inequality, we have

$$\begin{aligned} |W_1| + |\left( e_t,1\right) _j| \le C h^{\frac{1}{2}}( {\Vert {\xi }\Vert _{I_j}} + {\Vert {\xi _x}\Vert _{I_j}} + {\Vert {\xi _t}\Vert _{I_j}} + {\Vert {\eta _t}\Vert _{I_j}} ). \end{aligned}$$

By the definition of the projection $P_h^-$, (2.9b)

$$\begin{aligned} |W_3| = 0. \end{aligned}$$

Inserting the above two estimates into (3.41), we arrive at

$$\begin{aligned} | {[\![{\xi }]\!]}_{j - \frac{1}{2}}| \le C h^{\frac{1}{2}}( {\Vert {\xi }\Vert _{I_j}} + {\Vert {\xi _x}\Vert _{I_j}} + {\Vert {\xi _t}\Vert _{I_j}} + {\Vert {\eta _t}\Vert _{I_j}} ), \end{aligned}$$

which iswhere we have used the bound for ${\Vert {\xi }\Vert }$, the relation (3.4b) and approximation error estimates (2.10a), and thus (3.40) follows. Therefore, (3.37) is valid for $\alpha = 1$. $\square$

**Step 2**  Suppose that (3.37) is true for ${\alpha = \ell }$. That is

3.42a $$\begin{aligned} {\Vert {\partial _h^{m} \xi }\Vert }&\le C h^{k+1},\quad \forall ~ 0\le m \le \ell - 1,\end{aligned}$$

3.42b $$\begin{aligned} {\Vert {(\partial _{\mathfrak M}^{\beta } \xi )_x}\Vert } + h^{-\frac{1}{2}} {\left| [{\partial _{\mathfrak M}^{\beta } \xi }]\right| }&\le C ({\Vert {\partial _h^{\beta _1} \partial _{t}^{\beta _2+1}\xi }\Vert } + h^{k+1}),\quad \forall ~ |\beta | = \beta _1 + \beta _2 \le \ell - 1,\end{aligned}$$

3.42c $$\begin{aligned} {\Vert {\partial _{\mathfrak M}^{\gamma } \xi }\Vert }&\le C h^{k+1},\quad \forall ~ |\gamma | \le \ell \quad and \quad \gamma \ne (\ell ,0), \end{aligned}$$

let us prove that it also holds for ${\alpha = \ell +1}$.

First, as shown in our claim, (3.42) implies that

$$\begin{aligned} {\Vert {\partial _h^{\ell } \xi (T)}\Vert } \le C h^{k+1}. \end{aligned}$$

The above estimate together with (3.42a) produces

3.43 $$\begin{aligned} {\Vert {\partial _h^{m} \xi }\Vert } \le C h^{k+1}, \quad \forall ~ 0\le m \le \ell . \end{aligned}$$

Therefore, (3.37a) is valid for $\alpha = \ell +1$.

Next, by assumption (3.42b), we can see that to show (3.37b) for $\alpha = \ell +1$, we need only to show

$$\begin{aligned} {\Vert {(\partial _{\mathfrak M}^{\beta } \xi )_x}\Vert } + h^{-\frac{1}{2}} {\left| [{\partial _{\mathfrak M}^{\beta } \xi }]\right| } \le C \left( {\Vert {\partial _h^{\beta _1} \partial _{t}^{\beta _2+1}\xi }\Vert } + h^{k+1}\right) ,\quad \forall ~ |\beta | = \ell . \end{aligned}$$

Without loss of generality, let us take $\beta = (\ell ,0)$ for example. To this end, we consider the following error equation

$$\begin{aligned} \left( \partial _h^{\ell } e_t,v_h\right) = {{\mathcal {H}}} (\partial _h^{\ell } (a \xi ),v_h) + {{\mathcal {H}}} (\partial _h^{\ell } (a {\eta }),v_h), \end{aligned}$$

which holds for any $v_h \in { V_{h}^\alpha }$. We use Leibniz rule (2.6b) to write out $\partial _h^{\ell } (a\xi )$ as

$$\begin{aligned} \partial _h^{\ell } \left( a \xi \right) = \sum _{i=0}^\ell \left( \begin{array}{c} \ell \\ i \end{array}\right) \partial _h^{i} a \left( x + \frac{\ell -i}{2} h \right) \partial _h^{\ell -i} \xi \left( x - \frac{i}{2} h \right) \triangleq \sum _{i=0}^\ell z_i. \end{aligned}$$

Therefore, the error equation becomes

3.44 $$\begin{aligned} \left( \partial _h^{\ell } e_t,v_h\right) = \sum _{i=0}^\ell Z_i + {{\mathcal {H}}} (\partial _h^{\ell } (a {\eta }),v_h), \end{aligned}$$

where $Z_i = {{\mathcal {H}}} (z_i,v_h)$ for $i = 0,\ldots , \ell$. Let us now work on $Z_0$. By the *strong* form of ${{\mathcal {H}}}$, (2.4b), we have

$$\begin{aligned} Z_0 = {{\mathcal {H}}} (a \partial _h^{\ell } \xi ,v_h) = - \left( (a \partial _h^{\ell } \xi )_x,v_h\right) - {\sum _{j = 1}^N}\left( a [\![{\partial _h^{\ell } \xi }]\!] v_h^+\right) _{j' - \frac{1}{2}}. \end{aligned}$$

Denote $L^k$ the standard Legendre polynomials of degree *k* in $[-1, 1]$. If we now let $v_h = (\partial _h^{\ell } \xi )_x - d L_k(s)$ with $d= (-1)^k \left( (\partial _h^{\ell } \xi )_x\right) _{j' - \frac{1}{2}}^+$ being a constant and $s = \frac{2(x-x_{j'})}{h}$, we get

$$\begin{aligned} Z_0&= - \left( a(x_{j'}) (\partial _h^{\ell } \xi )_x,v_h\right) - \left( (a(x) - a(x_{j'}))(\partial _h^{\ell } \xi )_x,v_h\right) - \left( a_x \partial _h^{\ell } \xi ,v_h\right) \\&\triangleq - Z_{0,0} - Z_{0,1} - Z_{0,2}, \end{aligned}$$

since $(v_h)_{j' -\frac{1}{2}}^+ = 0$. Substituting above expression into (3.44) and taking into account the assumption that $a(x) \ge \delta >0$, we have

3.45 $$\begin{aligned} \delta {\Vert {(\partial _h^{\ell } \xi )_x}\Vert ^2} \le Z_{0,0} = \sum _{i = 1}^\ell Z_i + {{\mathcal {H}}} (\partial _h^{\ell } (a{\eta }),v_h) - Z_{0,1} - Z_{0,2} - \left( {\partial _h^{\ell } e}_t,v_h\right) . \end{aligned}$$

It is easy to show by Corollary 1 that

3.46a $$\begin{aligned} \left| \sum _{i = 1}^\ell Z_i \right| \le C \sum _{i = 1}^\ell \left( {\Vert {\partial _h^{\ell -i} \xi }\Vert } + {\Vert {(\partial _h^{\ell -i} \xi )_x}\Vert } + h^{\frac{1}{2}}{\left| [{\partial _h^{\ell -i} \xi }]\right| } \right) {\Vert {v_h}\Vert } \le C h^{k+1}{\Vert {v_h}\Vert },\nonumber \\ \end{aligned}$$

where we have used (3.42a)–(3.42c), since $\ell - i \le \ell -1$ for $i\ge 1$. By Corollary 2, we have

3.46b $$\begin{aligned} {{\mathcal {H}}} (\partial _h^{\ell } (a{\eta }),v_h) \le C h^{k+1}{\Vert {v_h}\Vert }. \end{aligned}$$

By (3.43) and inverse property (i), we arrive at a bound for $Z_{0,1}$ and $Z_{0,2}$

3.46c $$\begin{aligned} |Z_{0,1}| + |Z_{0,2}| \le C {\Vert {\partial _h^{\ell } \xi }\Vert } {\Vert {v_h}\Vert } \le C h^{k+1}{\Vert {v_h}\Vert }. \end{aligned}$$

The triangle inequality and the approximation error estimate (3.3) yield

3.46d $$\begin{aligned} \left| \left( {\partial _h^{\ell } e}_t,v_h\right) \right| \le C \left( {\Vert {\partial _h^{\ell } \partial _{t} \xi }\Vert } + h^{k+1}\right) {\Vert {v_h}\Vert }. \end{aligned}$$

Collecting the estimates (3.46a)–(3.46d) into (3.45) and using the fact that ${\Vert {v_h}\Vert } \le C {\Vert {(\partial _h^{\ell } \xi )_x}\Vert }$, we arrive at

3.47 $$\begin{aligned} {\Vert {(\partial _h^{\ell } \xi )_x}\Vert }\le C ({\Vert {\partial _h^{\ell } \partial _{t} \xi }\Vert } + h^{k+1}). \end{aligned}$$

If we take $v_h = 1$ in the cell error equation and use an analysis similar to that in the proof of (3.40), we will get the following relation

3.48 $$\begin{aligned} h^{-\frac{1}{2}} {\left| [{\partial _h^{\ell } \xi }]\right| }\le C ({\Vert {\partial _h^{\ell } \partial _{t} \xi }\Vert } + h^{k+1}). \end{aligned}$$

A combination of (3.47) and (3.48) gives us

$$\begin{aligned} {\Vert {(\partial _h^{\ell } \xi )_x}\Vert }+ h^{-\frac{1}{2}} {\left| [{\partial _h^{\ell } \xi }]\right| }\le C ({\Vert {\partial _h^{\ell } \partial _{t} \xi }\Vert } + h^{k+1}). \end{aligned}$$

Therefore, (3.37b) still holds for $\alpha = \ell +1$.

Finally, let us verify that (3.37c) is valid for $\alpha = \ell + 1$. Noting the assumption (3.42c), we need only consider $|\gamma | = \ell +1$ and $\gamma \ne (\ell +1,0)$. To do that, we start from the estimate for ${\Vert {\partial _{\mathfrak M}^{\gamma } \xi }\Vert }$ with $\gamma = (0, \ell +1)$ that has already been established in (3.33). By an analysis similar to that in the proof of Lemma 8 and taking into account relations (3.37a) and (3.37b) for $\alpha = \ell +1$, we conclude that (3.37c) is valid for $\gamma = (1,\ell )$. Repeating the above procedure, we can easily verify that (3.37c) is also valid for $\gamma = (2, \ell -1),\ldots ,(\ell ,1)$. Therefore, (3.37c) holds true for $\alpha = \ell +1$, and thus (3.34) in Theorem 3 is valid for general $\alpha \ge 1$.

## Superconvergent error estimates

For nonlinear hyperbolic equations, the negative-order norm estimate of the DG error itself has been established in [16]. However, by post-processing theory [5, 11], negative-order norm estimates of divided differences of the DG error are also needed to obtain superconvergent error estimates for the post-processed solution in the $L^2$ norm. Using a duality argument together with $L^2$ norm estimates established in Sect. 3, we show that for a given time *T*, the $\alpha$-th order divided difference of the DG error in the negative-order norm achieves $\left( {2k + \frac{3}{2} - \frac{\alpha }{2}}\right)$th order superconvergence. As a consequence, the DG solution $u_h(T)$, converges with at least $\left( {\frac{3}{2}k+1}\right)$th order in the $L^2$ norm when convolved with a particularly designed kernel.

We are now ready to state our main theorem about the negative-order norm estimates of divided differences of the DG error.

### Theorem 4

For any $1 \le \alpha \le k+1$, let $\partial _h^{\alpha } u$ be the exact solution of the problem (2.1), which is assumed to be sufficiently smooth with bounded derivatives, and assume that $|f'(u)|$ is uniformly lower bounded by a positive constant. Let $\partial _h^{\alpha } u_h$ be the numerical solution of scheme (2.2) with initial condition $\partial _h^{\alpha } u_h(0) = {{{\mathbb {Q}}}_h} (\partial _h^{\alpha } u_0)$ when the upwind flux is used. For a uniform mesh of $\Omega = (a,b)$, if the finite element space ${ V_{h}^\alpha }$ of piecewise polynomials with arbitrary degree $k \ge 1$ is used, then for small enough *h* and any $T > 0$ there holds the following error estimate

4.1 $$\begin{aligned} \Vert {\partial _h^{\alpha } (u - u_h)(T)}\Vert _{-{(k+1)},\Omega }\le C h^{2k + \frac{3}{2} - \frac{\alpha }{2}}, \end{aligned}$$

where the positive constant *C* depends on *u*, $\delta$, *T* and *f*, but is independent of *h*.

Combining Theorems 4 and 1, we have

### Corollary 5

Under the same conditions as in Theorem 4, if in addition $K_h^{\nu ,k+1}$ is a convolution kernel consisting of $\nu = 2k+1 + \omega$ $(\omega \ge \lceil - \frac{k}{2} \rceil )$ B-splines of order $k+1$ such that it reproduces polynomials of degree $\nu - 1$, then we have

4.2 $$\begin{aligned} {\Vert {u - u_h^\star }\Vert } \le C {h^{\frac{3}{2}k+1}}, \end{aligned}$$

where $$u_h^\star = K_h^{\nu ,k+1} \star u_h$$.

### Remark 6

The $({\frac{3}{2}k+1})$th order superconvergence is shown for the negative ${k+1}$ norm, and thus is valid for B-splines of order ${k+1}$ (by Theorem 1). For general order of B-splines $\ell$ and $\alpha \le \ell$, using similar argument for the proof of the negative ${k+1}$ norm estimates (see Sect. 4.1), we can prove the following superconvergent error estimate

$$\begin{aligned} \Vert {\partial _h^{\alpha } (u - u_h)(T)}\Vert _{-{\ell },\Omega } \le C h^{{k + \frac{3}{2} - \frac{\alpha }{2}}+ \ell -1} \le C h^{k+\frac{\ell +1}{2}}. \end{aligned}$$

Therefore, from the theoretical point of view, a higher order of B-splines $\ell$ may lead to a superconvergence result of higher order, for example $\ell = k+1$ and thus $({\frac{3}{2}k+1})$th order in Corollary 5. However, from the practical point of view, changing the order of B-splines does not affect the order of superconvergence; see Sect. 5 below and also [17].

### Proof of the main results in the negative-order norm

Similar to the proof for the $L^2$ norm estimates of the divided differences in Sect. 3.2, we will only consider the case $f'(u(x,t)) \ge \delta > 0$ for all $(x,t) \in \Omega \times [0,T]$. To perform the analysis for the negative-order norm, by (2.5), we need to concentrate on the estimate of

4.3 $$\begin{aligned} \left( \partial _h^{\alpha } (u-u_h)(T),\Phi \right) \end{aligned}$$

for $\Phi \in C_0^\infty (\Omega )$. To do that, we use the duality argument, following [16, 19]. For the nonlinear hyperbolic Eq. (2.1), we choose the dual equation as: Find a function $\varphi$ such that $\varphi (\cdot ,t)$ is periodic for all $t \in [0, T]$ and

4.4a $$\begin{aligned} \partial _h^{\alpha } \varphi _t + f'(u) \partial _h^{\alpha } \varphi _x&= 0, \quad (x, t)\in \Omega \times [0, T),\end{aligned}$$

4.4b $$\begin{aligned} \varphi (x,T)&=\Phi (x), \quad x \in \Omega . \end{aligned}$$

Unlike the purely linear case [11, 15] or the variable coefficient case [19], the dual equations for nonlinear problems will no longer preserve the inner product of original solution $\partial _h^{\alpha } u$ and its dual solution $\varphi$, namely $\frac{d}{dt}\left( \partial _h^{\alpha } u,\varphi \right) \ne 0$. In fact, if we multiply (2.1a) by $\varphi$ and (4.4a) by $(-1)^{\alpha } u$ and integrate over $\Omega$, we get, after using integration by parts and summation by parts (2.6d), that

4.5 $$\begin{aligned} \frac{d}{dt}\left( \partial _h^{\alpha } u,\varphi \right) + {\mathcal {F}}(u;\varphi ) = 0, \end{aligned}$$

where

$$\begin{aligned} {\mathcal {F}}(u;\varphi ) = (-1)^{\alpha } \left( f'(u) u - f(u) ,\partial _h^{\alpha } \varphi _x\right) . \end{aligned}$$

Note that ${\mathcal {F}}(u;\varphi )$ is the same as that in [16] when $\alpha = 0$. We now integrate (4.5) with respect to time between 0 and *T* to obtain a relation $\left( \partial _h^{\alpha } u,\varphi \right)$ in different time level

4.6 $$\begin{aligned} \left( \partial _h^{\alpha } u,\varphi \right) (T) = \left( \partial _h^{\alpha } u,\varphi \right) (0) - {\int _{0}^T{{\mathcal {F}}(u;\varphi )}dt }. \end{aligned}$$

In what follows, we work on the estimate of (4.3). To do that, let us begin by using the relation (4.6) to get an equivalent form of (4.3). It reads, for any $\chi \in { V_{h}^\alpha }$

$$\begin{aligned}&\left( \partial _h^{\alpha } (u-u_h)(T),\Phi \right) \nonumber \\&\quad = \left( \partial _h^{\alpha } (u-u_h)(T),\varphi (T)\right) \nonumber \\&\quad = \left( \partial _h^{\alpha } u,\varphi \right) (0) - {\int _{0}^T{{\mathcal {F}}(u;\varphi )}dt } - \left( \partial _h^{\alpha } u_h,\varphi \right) (0) - {\int _{0}^T{\frac{d}{dt}\left( \partial _h^{\alpha } u_h,\varphi \right) }dt } \nonumber \\&\quad = \left( \partial _h^{\alpha } (u - u_h),\varphi \right) (0) - {\int _{0}^T{\left( \left( {(\partial _h^{\alpha } u_h)}_t,\varphi \right) + \left( \partial _h^{\alpha } u_h,\varphi _t\right) \right) }dt } - {\int _{0}^T{{\mathcal {F}}(u;\varphi )}dt } \nonumber \\&\quad = {\mathbb {G}}_1 + {\mathbb {G}}_2 + {\mathbb {G}}_3, \nonumber \end{aligned}$$

where

$$\begin{aligned} {\mathbb {G}}_1&= \left( \partial _h^{\alpha } (u - u_h),\varphi \right) (0), \\ {\mathbb {G}}_2&= - {\int _{0}^T{\left( \left( \partial _h^{\alpha } {u_h}_t,\varphi - \chi \right) - {{\mathcal {H}}} (\partial _h^{\alpha } f(u_h),\varphi - \chi ) \right) }dt }, \\ {\mathbb {G}}_3&= - {\int _{0}^T{\left( \left( \partial _h^{\alpha } u_h,\varphi _t\right) + {{\mathcal {H}}} (\partial _h^{\alpha } f(u_h),\varphi ) + {\mathcal {F}}(u,\varphi )\right) }dt } \end{aligned}$$

will be estimated one by one below.

Note that in our analysis for ${\Vert {\partial _h^{\alpha } (u - u_h)(T)}\Vert }$ in Theorem 2, we need to choose a particular initial condition, namely $\partial _h^{\alpha } u_h(0) = P_h^- {(\partial _h^{\alpha } u_0)}$ instead of $\partial _h^{\alpha } u_h(0) = P_k {(\partial _h^{\alpha } u_0)}$ for purely linear equations [11, 15]. Thus, we arrive at a slightly different bound for ${\mathbb {G}}_1$, as shown in the following lemma. We note that using the $L^2$ projection in the numerical examples is still sufficient to obtain superconvergence.

#### Lemma 10

(Projection estimate) There exists a positive constant *C*, independent of *h*, such that

4.7 $$\begin{aligned} |{\mathbb {G}}_1| \le C h^{2k+1}\Vert {\partial _h^{\alpha } u_0}\Vert _{{k+1}} \Vert {\varphi (0)}\Vert _{{k+1}}. \end{aligned}$$

#### Proof

Since $\partial _h^{\alpha } u_h(0) = P_h^- {(\partial _h^{\alpha } u_0})$, we have the following identity

$$\begin{aligned} {\mathbb {G}}_1 = \left( \partial _h^{\alpha } (u - u_h),\varphi \right) (0) = \left( \partial _h^{\alpha } u_0 - P_h^- (\partial _h^{\alpha } u_0),\varphi (0) - P_{k-1} \varphi (0)\right) , \end{aligned}$$

where $P_{k-1}$ is the $L^2$ projection into $V_h^{k-1}$. A combination of Cauchy–Schwarz inequality and approximation error estimates (2.10a) leads to the desired result (4.7). $\square$

The bound for ${\mathbb {G}}_2$ is given in the following lemma.

#### Lemma 11

(Residual) There exists a positive constant *C*, independent of *h*, such that

4.8 $$\begin{aligned} |{\mathbb {G}}_2| \le C h^{2k + \frac{3}{2} - \frac{\alpha }{2}}\Vert {\varphi } \Vert _{L^1([0,T];H^{k+1})}. \end{aligned}$$

#### Proof

Denoting by *G* the term inside the time integral of ${\mathbb {G}}_2$, we get, by taking $\chi = P_k \varphi$, the following expression for *G*,

$$\begin{aligned} G = - {{\mathcal {H}}} (\partial _h^{\alpha } f(u_h),\varphi - P_{k} \varphi ), \end{aligned}$$

which is equivalent to

$$\begin{aligned} G&= - \left( \partial _h^{\alpha } (f(u_h)- f(u)),(\varphi - P_{k} \varphi )_x\right) + \left( \partial _h^{\alpha } f(u)_x,\varphi - P_{k} \varphi \right) \\&\quad ~ \!+ {\sum _{j = 1}^N}\left( \partial _h^{\alpha } (f(u) - f(u_h^-)) [\![{\varphi - P_{k} \varphi }]\!] \right) _{j' - \frac{1}{2}}\\&\triangleq G_1 + G_2 + G_3, \end{aligned}$$

where we have added and subtracted the term $\left( \partial _h^{\alpha } f(u),(\varphi - P_{k} \varphi )_x\right)$ and used integration by parts.

Let us now consider the estimates of $G_1, G_2, G_3$. For $G_1$, by using the second order Taylor expansion for $f(u) - f(u_h)$, (3.9), we get

$$\begin{aligned} G_1&= \left( \partial _h^{\alpha } \left( f'(u)e - R_1 e^2\right) ,(\varphi - P_{k} \varphi )_x\right) \\&= \left( \partial _h^{\alpha } (f'(u)e),(\varphi - P_{k} \varphi )_x\right) - \left( \partial _h^{\alpha } (R_1 e^2),(\varphi - P_{k} \varphi )_x\right) \\&\triangleq G_1^{lin } - G_1^{nlr }, \end{aligned}$$

where $G_1^{lin }$ and $G_1^{nlr }$, respectively, represent the linear part and the nonlinear part of $G_1$. It is easy to show, by using the Leibniz rule (2.6b) and Cauchy–Schwarz inequality, that

4.9a $$\begin{aligned} |G_1^{lin }|&\le C {\sum _{\ell = 0}^\alpha }{\Vert {\partial _h^{\alpha - \ell } e}\Vert } {\Vert {(\varphi - P_{k} \varphi )_x}\Vert } \nonumber \\&\le {C_{\star }}h^{2k + \frac{3}{2} - \frac{\alpha }{2}}\Vert {\varphi }\Vert _{{k+1}}, \end{aligned}$$

where we have used the estimate for ${\Vert {\partial _h^{\alpha - \ell } e}\Vert }$ in Corollary 3 and the approximation error estimate (2.10a). Analogously, for high order nonlinear term $G_1^{nlr }$, we have

4.9b $$\begin{aligned} |G_1^{nlr }|&\le C {\sum _{\ell = 0}^\alpha }{\Vert {\partial _h^{\alpha - \ell } e^2}\Vert } {\Vert {(\varphi - P_{k} \varphi )_x}\Vert } \nonumber \\&\le C {\sum _{m = 0}^\alpha }{\Vert {\partial _h^{m} e}\Vert _{\infty }} {\Vert {\partial _h^{\alpha - m} e}\Vert }{\Vert {(\varphi - P_{k} \varphi )_x}\Vert } \nonumber \\&\le {C_{\star }}h^{3k + \frac{5}{2} - \frac{\alpha }{2}}\Vert {\varphi }\Vert _{{k+1}}, \end{aligned}$$

where we have used the (2.6b) twice, the inverse property (iii), the $L^2$ norm estimate (3.2), and the approximation error estimate (2.10a). A combination of above two estimates yields

4.10 $$\begin{aligned} |G_1| \le {C_{\star }}h^{2k + \frac{3}{2} - \frac{\alpha }{2}} \Vert {\varphi }\Vert _{{k+1}}. \end{aligned}$$

To estimate $G_2$, we use an analysis similar to that in the proof of ${\mathbb {G}}_1$ in Lemma 10 and make use of the orthogonal property of the $L^2$ projection $P_k$ to get

4.11 $$\begin{aligned} G_2 = \left( \partial _h^{\alpha } f(u)_x- P_k(\partial _h^{\alpha } f(u)_x),\varphi - P_{k} \varphi \right) \le C h^{2k+2}\Vert {\partial _h^{\alpha } f(u)_x}\Vert _{{k+1}} \Vert {\varphi }\Vert _{{k+1}}, \end{aligned}$$

where we have used the approximation error estimate (2.10a).

We proceed to estimate $G_3$. It follows from the Taylor expansion (3.9), the Leibniz rule (2.6b), the Cauchy–Schwarz inequality and the inverse properties (ii), (iii) that

4.12 $$\begin{aligned} |G_3|&\le C {\sum _{\ell = 0}^\alpha }{\Vert {\partial _h^{\ell } e}\Vert _{\Gamma _{\!h}}} {\Vert {\varphi - P_{k} \varphi }\Vert _{\Gamma _{\!h}}} + {C_{\star }}{\sum _{m = 0}^\alpha }{\Vert {\partial _h^{m} e}\Vert _{\infty }} {\Vert {\partial _h^{\alpha - m} e}\Vert _{\Gamma _{\!h}}} {\Vert {\varphi - P_{k} \varphi }\Vert _{\Gamma _{\!h}}}\nonumber \\&\le C h^{2k + \frac{3}{2} - \frac{\alpha }{2}}\Vert {\varphi }\Vert _{{k+1}} + {C_{\star }}h^{3k + \frac{5}{2} - \frac{\alpha }{2}}\Vert {\varphi }\Vert _{{k+1}} \nonumber \\&\le C h^{2k + \frac{3}{2} - \frac{\alpha }{2}}\Vert {\varphi }\Vert _{{k+1}}, \end{aligned}$$

where we have also used (3.2) and (2.10a). Collecting the estimates (4.10)–(4.12), we get

4.13 $$\begin{aligned} |G| \le C h^{2k + \frac{3}{2} - \frac{\alpha }{2}} \Vert {\varphi }\Vert _{{k+1}}. \end{aligned}$$

Consequently, the estimate for ${\mathbb {G}}_2$ follows by integrating the above inequality with respect to time. $\square$

We move on to the estimate of ${\mathbb {G}}_3$, which is given in the following lemma.

#### Lemma 12

(Consistency) There exists a positive constant *C*, independent of *h*, such that

4.14 $$\begin{aligned} |{\mathbb {G}}_3| \le C h^{2k + 3 - \frac{\alpha }{2}} \Vert {\varphi } \Vert _{L^1([0,T];H^{k+1})}. \end{aligned}$$

#### Proof

To do that, let us denote by $G_4$ the term inside the integral ${\mathbb {G}}_3$ and take into account (2.6d) to obtain an equivalent form of $G_4$

$$\begin{aligned} G_4&= (-1)^{\alpha } \left( u_h, \partial _h^{\alpha } \varphi _t\right) + (-1)^{\alpha } \left( f(u_h),\partial _h^{\alpha } \varphi _x\right) + (-1)^{\alpha } \left( f'(u) u - f(u) ,\partial _h^{\alpha } \varphi _x\right) \\&\quad ~ \!+ {\sum _{j = 1}^N}\left( \partial _h^{\alpha } f(u_h^-) [\![{\varphi }]\!]\right) _{j'+\frac{1}{2}} \\&= (-1)^{\alpha } \left( f(u_h) - f(u) - f'(u)(u_h - u),\partial _h^{\alpha } \varphi _x\right) , \end{aligned}$$

where we have used the dual problem (4.4) and the fact that $[\![{\varphi }]\!]= 0$ due to the smoothness of $\varphi$. Next, by using the second order the Taylor expansion (3.9) and (2.6d) again, we arrive at

$$\begin{aligned} G_4 = \left( \partial _h^{\alpha } (R_1 e^2),\varphi _x\right) . \end{aligned}$$

If we now use (2.6b) twice for $\partial _h^{\alpha } (R_1 e^2)$ and the Cauchy–Schwarz inequality together with the error estimate (3.2), we get

4.15 $$\begin{aligned} |G_4|&\le C {\sum _{\ell = 0}^\alpha }{\sum _{m = 0}^{ \ell }}{\Vert {\partial _h^{m} e}\Vert } {\Vert {\partial _h^{ \ell - m} e}\Vert } {\Vert {\varphi _x}\Vert _{\infty }} \nonumber \\&\le {C_{\star }}h^{2k+3 -\frac{\alpha }{2}} \Vert {\varphi }\Vert _{{k+1}}, \end{aligned}$$

where we have also used the Sobolev inequality $${\Vert {\varphi _x}\Vert _{\infty }} \le C \Vert {\varphi }\Vert _{{k+1}}$$, under the condition that $$ k > 1/2$$. The bound for $${\mathbb {G}}_3$$ follows immediately by integrating the above inequality with respect to time. $$\square $$

We are now ready to obtain the final negative-order norm error estimates for the divided differences. By collecting the results in Lemmas 10–12 and taking into account the regularity result in Lemma 3, namely $\Vert {\varphi }\Vert _{{k+1}}\le C \Vert {\Phi }\Vert _{{k+1}}$, we get a bound for $\left( \partial _h^{\alpha } (u - u_h)(T),\Phi \right)$

$$\begin{aligned} \left( \partial _h^{\alpha } (u - u_h)(T),\Phi \right) \le C h^{2k + \frac{3}{2} - \frac{\alpha }{2}}\Vert {\Phi }\Vert _{{k+1}}. \end{aligned}$$

Thus, by (2.5), we have the bound for the negative-order norm

$$\begin{aligned} \Vert {\partial _h^{\alpha } (u - u_h)(T)}\Vert _{-{(k+1)},\Omega } \le C h^{2k + \frac{3}{2} - \frac{\alpha }{2}}. \end{aligned}$$

This finishes the proof of Theorem 4.

**Table 1:** Before post-processing (left), after post-processing (middle) and post-processing with the more compact kernel (right). T = 0.3 $L^2$- and $L^\infty$ errors for Example 1

Mesh	Before post-processing				Post-processed ($\omega = 0)$				Post-processed ($\omega = -2$)			
	$L^2$ error	Order	$L^\infty$ error	Order	$L^2$ error	Order	$L^\infty$ error	Order	$L^2$ error	Order	$L^\infty$ error	Order
$$P^2$$												
20	1.54E−04	–	5.70E−04	–	1.04E−04	–	3.16E−04	–	5.36E−04	–	1.40E−03	–
40	2.06E−05	2.90	1.03E−04	2.47	2.28E−06	5.52	7.53E−06	5.39	3.69E−05	3.86	9.93E−05	3.81
80	2.73E−06	2.92	1.55E−05	2.73	3.97E−08	5.84	1.38E−07	5.77	2.37E−06	3.96	6.43E−06	3.95
160	3.56E−07	2.93	2.25E−06	2.78	1.13E−09	5.13	9.86E−09	3.81	1.49E−07	3.99	4.06E−07	3.99
$$P^3$$												
20	7.68E−06	–	2.91E−05	–	5.88E−05	–	1.88E−04	–	1.59E−04	–	4.80E−04	–
40	5.21E−07	3.88	2.36E−06	3.62	5.47E−07	6.75	1.97E−06	6.58	3.71E−06	5.42	1.21E−05	5.31
80	3.45E−08	3.92	1.74E−07	3.76	2.87E−09	7.57	1.09E−08	7.50	6.56E−08	5.82	2.20E−07	5.78
160	2.23E−09	3.95	1.19E−08	3.87	1.22E−11	7.88	4.70E−11	7.86	1.06E−09	5.95	3.58E−09	5.94

## Numerical examples

For nonlinear hyperbolic equations, we proved $L^2$ norm superconvergence results of order ${\frac{3}{2}k+1}$ for post-processed errors, as shown in Corollary 5. The superconvergence results together with the post-processing theory by Bramble and Schatz in Theorem 1 entail us to design a more compact kernel to achieve the desired superconvergence order. We note that superconvergence of post-processed errors using the standard kernel (a kernel function composed of a linear combination of $2k+1$ B-splines of order $k+1$) for nonlinear hyperbolic equations has been numerically studied in [11, 16]. Note that the order of B-splines does not have significant effect on the rate of convergence numerically and that it is the number of B-splines that has greater effect to the convergence order theoretically [11], we will only focus on the effect of different total numbers (denoted by $\nu = 2k+1 + \omega$ with $\omega \ge \lceil - \frac{k}{2} \rceil$) of B-splines of the kernel in our numerical experiments. For more numerical results using different orders of B-splines, we refer the readers to [17].

We consider the DG method combined with the third-order Runge–Kutta method in time. We take a small enough time step such that the spatial errors dominate. We present the results for $P^2$ and $P^3$ polynomials only to save space, in which a specific value of $\omega$ is chosen to match the orders given in Corollary 5. For the numerical initial condition, we take the standard $L^2$ projection of the initial condition and we have observed little difference if the ${{{\mathbb {Q}}}_h}$ projection is used instead. Uniform meshes are used in all experiments. Only one-dimensional scalar equations are tested, whose theoretical results are covered in our main theorems.

### Example 1

We consider the Burgers quation on the domain $\Omega = (0, 2 \pi )$

5.1 $$\begin{aligned} {\left\{ \begin{array}{ll} u_t+\left( \frac{u^2}{2}\right) _x=0,\\ u(x,0)= \sin (x) \end{array}\right. } \end{aligned}$$

with periodic boundary conditions.

**Fig. 1:**
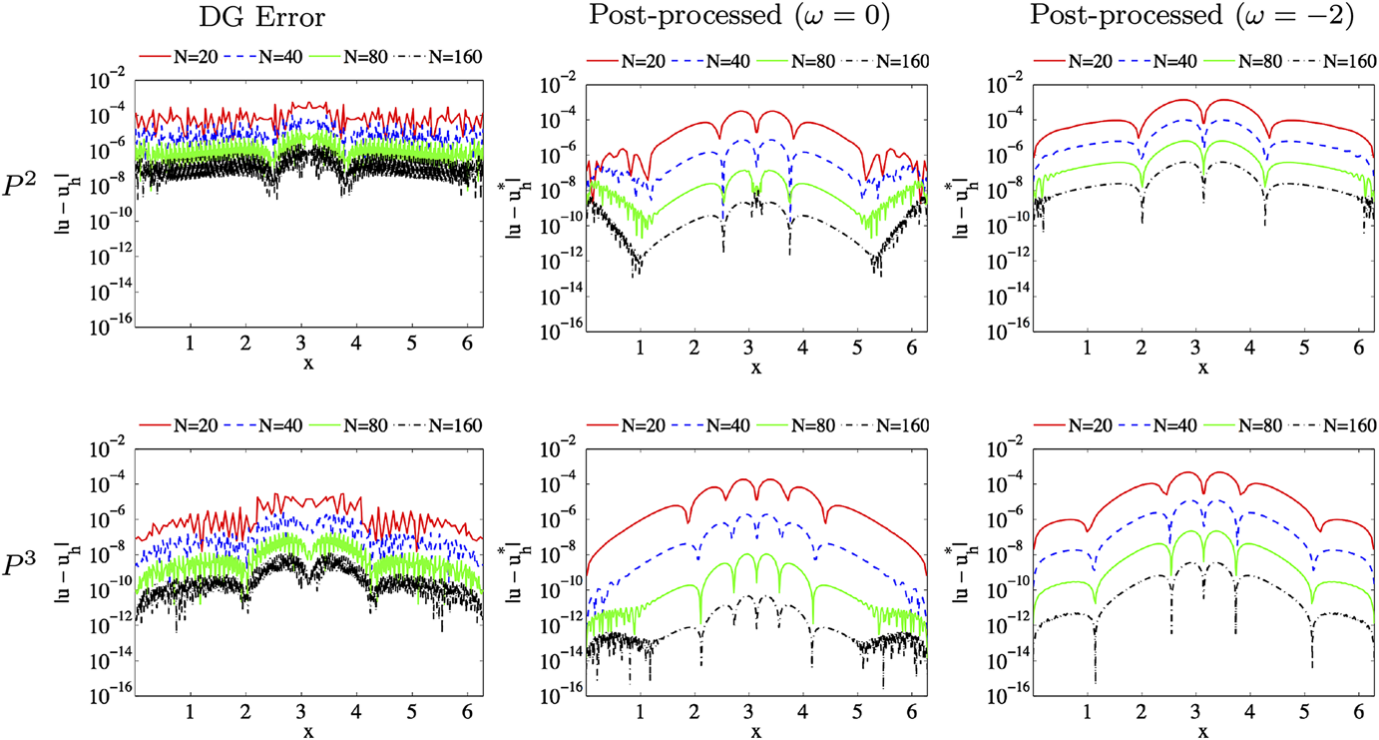
The errors in absolute value and in logarithmic scale for $P^2$ (*top*) and $P^3$ (*bottom*) polynomials with $N = 20, 40, 80$ and 160 elements for Example 1 where $f(u) = u^2/2$. Before post-processing (*left*), after post-processing (*middle*) and post-processing with the more compact kernel (*right*). $T = 0.3$

Noting that $f'(u)$ changes its sign in the computational domain, we use the Godunov flux, which is an upwind flux. The errors at $T = 0.3$, when the solution is still smooth, are given in Table 1. From the table, we can see that one can improve the order of convergence from ${k+1}$ to at least ${2k+1}$, which is similar to the results for Burgers equations in [11]. Moreover, superconvergence of order 2*k* can be observed for the compact kernel with $\omega = -2$, as, in general, a symmetric kernel could yield one additional order. This is why instead of $\omega = \lceil - \frac{k}{2} \rceil = -1$, $\omega = -2$ is chosen in our kernel. The pointwise errors are plotted in Fig. 1, which show that the post-processed errors are less oscillatory and much smaller in magnitude for a large number of elements as observed in [11], and that the errors of our more compact kernel with $\omega = -2$ are less oscillatory than that for the standard kernel with $\omega = 0$, although the magnitude of the errors increase. This example demonstrates that the superconvergence result also holds for conservation laws with a general flux function.

**Table 2:** Before post-processing (left), after post-processing (middle) and post-processing with the more compact kernel (right). T = 0.1 $L^2$- and $L^\infty$ errors for Example 2

Mesh	Before post-processing				Post-processed ($\omega = 0)$				Post-processed ($\omega = -2$)			
	$L^2$ error	Order	$L^\infty$ error	Order	$L^2$ error	Order	$L^\infty$ error	Order	$L^2$ error	Order	$L^\infty$ error	Order
$$P^2$$												
20	1.25E−04	–	5.76E−04	–	4.45E−05	–	1.61E−04	–	2.49E−04	–	7.98E−04	–
40	1.61E−05	2.95	7.64E−05	2.91	1.01E−06	5.46	4.03E−06	5.32	1.68E−05	3.88	5.73E−05	3.80
80	1.96E−06	3.04	1.02E−05	2.91	1.80E−08	5.81	7.35E−08	5.78	1.08E−06	3.97	3.72E−06	3.95
160	2.45E−07	3.00	1.32E−06	2.95	3.02E−10	5.90	1.25E−09	5.88	6.77E−08	3.99	2.35E−07	3.99
$$P^3$$												
20	3.99E−06	–	2.52E−05	–	2.50E−05	–	9.12E−05	–	6.64E−05	–	2.38E−04	–
40	2.62E−07	3.93	1.67E−06	3.91	2.41E−07	6.70	1.00E−06	6.51	1.57E−06	5.40	6.17E−06	5.27
80	1.68E−08	3.96	1.13E−07	3.89	1.29E−09	7.55	5.66E−09	7.47	2.79E−08	5.81	1.14E−07	5.76
160	1.04E−09	4.01	7.38E−09	3.93	5.45E−12	7.88	2.45E−11	7.85	4.51E−10	5.95	1.86E−09	5.94

### Example 2

In this example we consider the conservation laws with more general flux functions on the domain $\Omega = (0, 2 \pi )$

5.2 $$\begin{aligned} {\left\{ \begin{array}{ll} u_t+(e^u)_x=0,\\ u(x,0)= \sin (x) \end{array}\right. } \end{aligned}$$

with periodic boundary conditions.

**Fig. 2:**
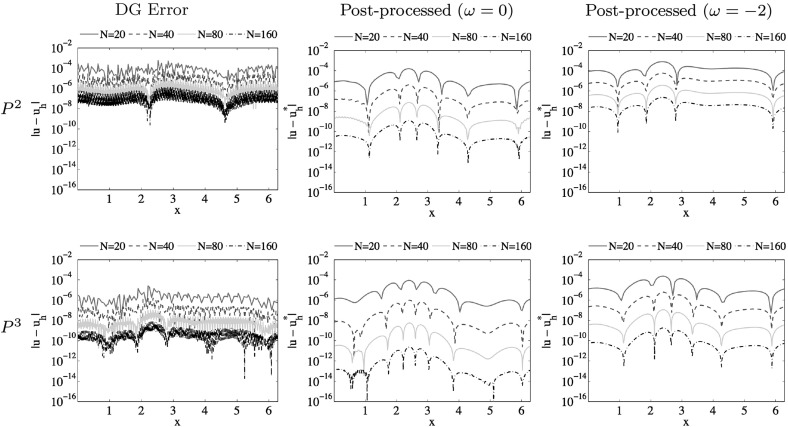
The errors in absolute value and in logarithmic scale for $P^2$ (*top*) and $P^3$ (*bottom*) polynomials with $N = 20, 40, 80$ and 160 elements for Example 2 where $f(u) = e^u$. Before post-processing (*left*), after post-processing (*middle*) and post-processing with the more compact kernel (*right*). $T = 0.1$

We test the Example 2 at $T = 0.1$ before the shock is developed. The orders of convergence with different kernels are listed in Table 2 and pointwise errors are plotted in Fig. 2. We can see that the post-processed errors are less oscillatory and much smaller in magnitude for most of elements as observed in [16], and that the errors of our more compact kernel with $\omega = -2$ are slightly less oscillatory than that for the standard kernel with $\omega = 0$. This example demonstrates that the accuracy-enhancement technique also holds true for conservation laws with a strong nonlinearity that is not a polynomial of *u*.

## Concluding remarks

In this paper, the accuracy-enhancement of the DG method for nonlinear hyperbolic conservation laws is studied. We first prove that the $\alpha$-th order divided difference of the DG error in the $L^2$ norm is of order ${k + \frac{3}{2} - \frac{\alpha }{2}}$ when piecewise polynomials of degree *k* and upwind fluxes are used, provided that $|f'(u)|$ is uniformly lower bounded by a positive constant. Then, by a duality argument, the corresponding negative-order norm estimates of order ${2k + \frac{3}{2} - \frac{\alpha }{2}}$ are obtained, ensuring that the SIAC filter will achieve at least $({\frac{3}{2}k+1})$th order superconvergence. As a by-product, we show, for variable coefficient hyperbolic equations with $f(u) = a(x) u$, the optimal error estimates of order ${k+1}$ for the $L^2$ norm of divided differences of the DG error, provided that |*a*(*x*)| is uniformly lower bounded by a positive constant. Consequently, the superconvergence result of order ${2k+1}$ is obtained for the negative-order norm. Numerical experiments are given which show that using more compact kernels are less oscillatory and that the superconvergence property holds true for nonlinear conservation laws with general flux functions, indicating that the restriction on *f*(*u*) is artificial. Based on our numerical results we can see that these estimates are not sharp. However, they indicate that a more compact kernel can be used in obtaining superconvergence results.

Future work includes the study of accuracy-enhancement of the DG method for one-dimensional nonlinear symmetric/symmetrizable systems and scalar nonlinear conservation laws in multi-dimensional cases on structured as well as unstructured meshes. Analysis of the superconvergence property of the local DG (LDG) method for nonlinear diffusion equations is also on-going work.

## Appendix

### The proof of Lemma 5

Let us prove the relation (3.24) in Lemma 5. Use the Taylor expansion (3.9) and the identity (2.6b) to rewrite $\partial _{h} {(f(u) - f(u_h))}$ as

$$\begin{aligned} \partial _{h} {(f(u) - f(u_h))}&= \partial _{h} {(f'(u)\xi )} + \partial _{h} {(f'(u){\eta })}- \partial _{h} {(R_1 e^2 )} \\&= f'(u(x+h/2))\bar{\xi }+ (\partial _{h} {f'(u)}) \xi (x-h/2) + \partial _{h} {(f'(u){\eta })} \\&\quad ~\! - R_1(u(x+h/2)) (\partial _{h} {e^2}) - (\partial _{h} {R_1}) e^2(x-h/2)\\&\triangleq \theta _1 + \cdots + \theta _5. \end{aligned}$$

This allows the error Eq. (3.6) to be written as

7.1 $$\begin{aligned} \left( \bar{e}_t,v_h\right) = \Theta _1 + \cdots + \Theta _5, \end{aligned}$$

with $\Theta _i = {{\mathcal {H}}} (\theta _i,v_h)$ $(i = 1,\ldots , 5)$. In what follows, we will estimate each term above separately.

First consider $\Theta _1$. Begin by using the *strong* form of ${{\mathcal {H}}}$, (2.4b), to get

$$\begin{aligned} \Theta _1 = {{\mathcal {H}}} (f'(u)\bar{\xi },v_h) = - \left( (f'(u)\bar{\xi })_x,v_h\right) - {\sum _{j = 1}^N}\left( f'(u)[\![{\bar{\xi }}]\!]v_h^+\right) _j. \end{aligned}$$

Next, let $L_k$ be the standard Legendre polynomial of degree *k* in $[-1,1]$, so $L_k(-1) = (-1)^k$, and $L_k$ is orthogonal to any polynomials of degree at most $k-1$. If we now let $v_h = \bar{\xi }_x - b L_k(s)$ with $b = (-1)^k (\bar{\xi }_x)_j^+$ being a constant and $s = \frac{2(x-x_{j+1/2})}{h} \in [-1, 1]$, we arrive at

7.2 $$\begin{aligned} \Theta _1 = - \left( \partial _{x} f'(u)\bar{\xi },v_h\right) - \left( f'(u)\bar{\xi }_x,\bar{\xi }_x - b L_k(s)\right) \triangleq - X - Y, \end{aligned}$$

since $(v_h)_j^+ = 0$. On each element $I_{j'}= I_{j+\frac{1}{2}} = (x_j,x_{j+1})$, by the linearization $f'(u)= f'(u_{j+\frac{1}{2}}) + (f'(u)- f'(u_{j+\frac{1}{2}}))$ with $u_{j+\frac{1}{2}} = u(x_{j+\frac{1}{2}},t)$ and noting $\left( \bar{\xi }_x,L_k\right) _{j+\frac{1}{2}} = 0$, we arrive at an equivalent form of *Y*

7.3 $$\begin{aligned} Y = Y_1 + Y_2, \end{aligned}$$

where

$$\begin{aligned} Y_1&= {\sum _{j = 1}^N}f'(u_{j+\frac{1}{2}}){\Vert {\bar{\xi }_x}\Vert _{I_{j+\frac{1}{2}}}^2}, \\ Y_2&= \left( (f'(u)- f'(u_{j+\frac{1}{2}}))\bar{\xi }_x,\bar{\xi }_x - b L_k\right) . \end{aligned}$$

By the inverse property (ii), it is easy to show, for $v_h = \bar{\xi }_x - b L_k(s)$, that

$$\begin{aligned} {\Vert {v_h}\Vert } \le C {\Vert {\bar{\xi }_x}\Vert }. \end{aligned}$$

Plugging above results into (7.1) and using the assumption that $f'(u(x,t)) \ge \delta > 0$, we get

7.4 $$\begin{aligned} \delta {\Vert {\bar{\xi }_x}\Vert ^2} \le Y_1 = \sum _{i=2}^5 \Theta _i -X - Y_2 - \left( \bar{e}_t,\bar{\xi }_x - b L_k\right) . \end{aligned}$$

We shall estimate the terms on the right side of (7.4) one by one below.

For $\Theta _2$, by the *strong* form of ${{\mathcal {H}}}$, (2.4b), we have

$$\begin{aligned} \Theta _2 = - \left( (\partial _{h} {f'(u)} \xi )_x,v_h\right) - {\sum _{j = 1}^N}{\left( \partial _{h} {f'(u)}[\![{\xi }]\!]v_h^+ \right) _j } = - \left( (\partial _{h} {f'(u)} \xi )_x,v_h\right) , \end{aligned}$$

since $(v_h)_j^+ = 0$. Thus, by Cauchy–Schwarz inequality, we arrive at a bound for $\Theta _2$

7.5a $$\begin{aligned} |\Theta _2| \le {C_{\star }}({\Vert {\xi }\Vert }+ {\Vert {\xi _x}\Vert }) {\Vert {\bar{\xi }_x}\Vert }. \end{aligned}$$

A direct application of Corollary 2 leads to a bound for $\Theta _3$

7.5b $$\begin{aligned} |\Theta _3| \le {C_{\star }}h^{k+1}{\Vert {\bar{\xi }_x}\Vert }. \end{aligned}$$

By using analysis similar to that in the proof of (3.13), we get

7.5c $$\begin{aligned} |\Theta _4|&\le {C_{\star }}h^{-1} {\Vert {e}\Vert _{\infty }} ({\Vert {\bar{\xi }}\Vert }+ h^{k+1}){\Vert {\bar{\xi }_x}\Vert }, \end{aligned}$$

7.5d $$\begin{aligned} |\Theta _5|&\le {C_{\star }}h^{-1} {\Vert {e}\Vert _{\infty }} ({\Vert {\xi }\Vert }+ h^{k+1}){\Vert {\bar{\xi }_x}\Vert }. \end{aligned}$$

By the Cauchy–Schwarz inequality, we have

7.5e $$\begin{aligned} |X| \le {C_{\star }}{\Vert {\bar{\xi }}\Vert }{\Vert {\bar{\xi }_x}\Vert }. \end{aligned}$$

Using the Cauchy–Schwarz inequality again together with the inverse property (i), and taking into account the fact that $|f'(u)- f'(u_{j+\frac{1}{2}})| \le {C_{\star }}h$ on each element $I_{j+\frac{1}{2}}$, we obtain

7.5f $$\begin{aligned} |Y_2| \le {C_{\star }}{\Vert {\bar{\xi }}\Vert }{\Vert {\bar{\xi }_x}\Vert }. \end{aligned}$$

The triangle inequality and the approximation error estimate (3.3) yield that

7.5g $$\begin{aligned} |\left( \bar{e}_t,v_h\right) | \le C ({\Vert {\bar{\xi }_t}\Vert } + h^{k+1}) {\Vert {\bar{\xi }_x}\Vert }. \end{aligned}$$

Finally, the error estimate (3.24) follows by collecting the estimates (7.5a)–(7.5g) into (7.4) and by using the estimates (3.4a)–(3.4c), (3.17) and (3.14). This finishes the proof of Lemma 5.

### The proof of Lemma 7

To prove the error estimate (3.26), it is necessary to get a bound for the initial error ${\Vert {\xi _{tt}(0)}\Vert }$. To do that, we start by noting that $\xi (0) = 0$, and that ${\Vert {\xi _t(0)}\Vert } \le C h^{k+1}$, which have already been proved in [18, Appendix A.2]. Next, note also that the first order time derivative of the original error equation

$$\begin{aligned} \left( e_{tt},v_h\right) = {{\mathcal {H}}} (\partial _{t} (f(u)- f(u_h)),v_h) \end{aligned}$$

still holds at $t=0$ for any $v_h \in { V_{h}^\alpha }$. If we now let $v_h = \xi _{tt}(0)$ and use a similar argument for the proof of ${\Vert {\xi _t(0)}\Vert }$ in [18], we arrive at a bound for ${\Vert {\xi _{tt}(0)}\Vert }$

7.6 $$\begin{aligned} {\Vert {\xi _{tt}(0)}\Vert } \le C h^{k+1}. \end{aligned}$$

We then move on to the estimate of ${\Vert {\xi _{tt}(T)}\Vert }$ for $T > 0$. To this end, we take the second order derivative of the original error equation with respect to *t* and let $v_h = \xi _{tt}$ to get

$$\begin{aligned} \left( e_{ttt},\xi _{tt}\right) = {{\mathcal {H}}} (\partial _{tt} (f(u)- f(u_h)),\xi _{tt}), \end{aligned}$$

which is

7.7 $$\begin{aligned} \frac{1}{2}\frac{d}{dt} {\Vert {\xi _{tt}}\Vert ^2}+ \left( {\eta }_{ttt},\xi _{tt}\right) = {{\mathcal {H}}} (\partial _{tt} (f(u)- f(u_h)),\xi _{tt}). \end{aligned}$$

To estimate the right-hand side of (7.7), we use the Taylor expansion (3.9) and the Leibniz rule for partial derivatives to rewrite $\partial _{tt} (f(u)- f(u_h))$ as

$$\begin{aligned} \partial _{tt} (f(u) - f(u_h))&= \partial _{tt} (f'(u)\xi ) + \partial _{tt} (f'(u){\eta })- \partial _{tt} (R_1 e^2 ) \\&= (\partial _{tt} f'(u)) \xi + 2 (\partial _{t} f'(u)) \xi _t+ f'(u)\xi _{tt}+ (\partial _{tt} f'(u)) {\eta }\\&\quad +\, 2 (\partial _{t} f'(u)) \eta _t+ f'(u)\eta _{tt}- (\partial _{tt} R_1)e^2 \\&\quad - 2 (\partial _{t} R_1) \partial _{t} e^2 - R_1 (\partial _{tt} e^2) \\&\triangleq \lambda _1 + \cdots + \lambda _9. \end{aligned}$$

Therefore, the right side of (7.7) can be written as

7.8 $$\begin{aligned} {{\mathcal {H}}} (\partial _{tt} (f(u)- f(u_h)),\xi _{tt}) = \Lambda _1 + \cdots + \Lambda _9 \end{aligned}$$

with $\Lambda _i = {{\mathcal {H}}} (\lambda _i,\xi _{tt})$ $(i = 1,\ldots , 9)$, which will be estimated one by one below.

By (2.11a) in Lemma 1, it is easy to show for $\Lambda _1$ that

7.9a

where we have used the estimates (3.4a)–(3.4c) and also Young’s inequality. Analogously,

7.9b

where we have also used the estimate (3.4c) and the relation (3.25). A direct application of (2.11b) in Lemma 1 together with the assumption that $f'(u) \ge \delta >0$ leads to the estimate for $\Lambda _3$:

7.9c

Noting that $\eta _t = u_t - P_h^- (u_t)$ and ${\eta }_{tt} = u_{tt} - P_h^- (u_{tt})$, we have, by Lemma 2

7.9d $$\begin{aligned} |\Lambda _4|+ |\Lambda _5|+|\Lambda _6| \le {C_{\star }}h^{k+1}{\Vert {\xi _{tt}}\Vert }. \end{aligned}$$

By an analysis similar to that in the proof of (3.13), we get

$$\begin{aligned} |\Lambda _7|&\le {C_{\star }}h^{-1} {\Vert {e}\Vert _{\infty }} ({\Vert {\xi }\Vert }+ h^{k+1}){\Vert {\xi _{tt}}\Vert }, \\ |\Lambda _8|&\le {C_{\star }}h^{-1} {\Vert {e}\Vert _{\infty }} ({\Vert {\xi _t}\Vert }+ h^{k+1}){\Vert {\xi _{tt}}\Vert }, \\ |\Lambda _9|&\le {C_{\star }}h^{-1} ({\Vert {e}\Vert _{\infty }} + {\Vert {e_t}\Vert _{\infty }})({\Vert {\xi _t}\Vert }+ {\Vert {\xi _{tt}}\Vert }+ h^{k+1}) {\Vert {\xi _{tt}}\Vert }. \end{aligned}$$

Note that the result of Lemma 7 is used to prove the convergence result for the second order divided difference of the DG error, which implies that $k \ge 1$. Therefore, by using the inverse property (iii), the superconvergence result (3.4a), (3.4c), and the approximation error estimate (2.10b), we have for small enough *h*

$$\begin{aligned} {C_{\star }}h^{-1} {\Vert {e}\Vert _{\infty }}\le & {} {C_{\star }}h^{-1} ({\Vert {\xi }\Vert _{\infty }}+ {\Vert {{\eta }}\Vert _{\infty }}) \le {C_{\star }}h^k \le C,\\ {C_{\star }}h^{-1} {\Vert {e_t}\Vert _{\infty }}\le & {} {C_{\star }}h^{-1} ({\Vert {\xi _t}\Vert _{\infty }}+ {\Vert {\eta _t}\Vert _{\infty }}) \le {C_{\star }}h^{k-\frac{1}{2}} \le C, \end{aligned}$$

where *C* is a positive constant independent of *h*. Consequently,

7.9e $$\begin{aligned} |\Lambda _7|&\le C ({\Vert {\xi }\Vert }+ h^{k+1}){\Vert {\xi _{tt}}\Vert }, \end{aligned}$$

7.9f $$\begin{aligned} |\Lambda _8|&\le C ({\Vert {\xi _t}\Vert }+ h^{k+1}){\Vert {\xi _{tt}}\Vert }, \end{aligned}$$

7.9g $$\begin{aligned} |\Lambda _9|&\le C ({\Vert {\xi _t}\Vert }+ {\Vert {\xi _{tt}}\Vert }+ h^{k+1}) {\Vert {\xi _{tt}}\Vert }. \end{aligned}$$

Collecting the estimates (7.9a)–(7.9g) into (7.7) and (7.8), we get, after a straightforward application of Cauchy–Schwarz inequality and Young’s inequality, that

where we have used the estimates (3.4a) and (3.4c) for the last step. Now, we integrate the above inequality with respect to time between 0 and *T* and combine with the initial error estimate (7.6) to obtain

By the estimates (3.4a) and (3.4c) again, we arrive at

7.10

Finally, using Gronwall’s inequality gives us

7.11

which completes the proof of Lemma 7.

### The proof of Lemma 8

To prove the error estimate (3.27), it is necessary to get a bound for the initial error ${\Vert {{\bar{\xi }}_t(0)}\Vert }$. To do that, we start by noting that $\xi (0) = 0$, and thus $\bar{\xi }(0) = 0$, due to the choice of the initial data. Next, note also that the error equation (3.6) still holds at $t=0$ for any $v_h \in { V_{h}^\alpha }$. If we now let $v_h = {\bar{\xi }}_t(0)$ and use a similar argument for the proof of ${\Vert {\xi _t(0)}\Vert }$ in [18], we arrive at a bound for ${\Vert {{\bar{\xi }}_t(0)}\Vert }$

7.12 $$\begin{aligned} {\Vert {{\bar{\xi }}_t(0)}\Vert } \le C h^{k+1}. \end{aligned}$$

We then move on to the estimate of ${\Vert {{\bar{\xi }}_t(T)}\Vert }$ for $T > 0$. To obtain this, take the time derivative of the error Eq. (3.6) and let $v_h = {\bar{\xi }}_t$ to get

$$\begin{aligned} \left( \bar{e}_{tt},{\bar{\xi }}_t\right) = {{\mathcal {H}}} (\partial _{t} \partial _{h} {(f(u) - f(u_h))},{\bar{\xi }}_t), \end{aligned}$$

which is

7.13 $$\begin{aligned} \frac{1}{2}\frac{d}{dt} {\Vert {{\bar{\xi }}_t}\Vert ^2}+ \left( {\bar{\eta }}_{tt},{\bar{\xi }}_t\right) = {{\mathcal {H}}} (\partial _{t} \partial _{h} {(f(u) - f(u_h))},{\bar{\xi }}_t). \end{aligned}$$

To estimate the right-hand side of (7.13), we use the Taylor expansion (3.9) and the Leibniz rule (2.6b) to rewrite $\partial _{t} \partial _{h} {(f(u) - f(u_h))}$ as

$$\begin{aligned}&\partial _{t} \partial _{h} {(f(u) - f(u_h))} \\&\quad = \partial _{h} {\partial _{t} (f'(u)\xi )} + \partial _{h} {\partial _{t} (f'(u){\eta })}- \partial _{h} {\partial _{t} (R_1 e^2 )} \\&\quad = \partial _{h} {(\partial _{t} f'(u)\xi )} + \partial _{h} {(f'(u)\xi _t)} + \partial _{h} {(\partial _{t} f'(u){\eta })} + \partial _{h} {(f'(u)\eta _t)}\\&\quad - \partial _{h} {( {R_1} \partial _{t} e^2)} - \partial _{h} {(\partial _{t} R_1 e^2)} \\&\quad = \partial _{t} f'(u(x+h/2)) \bar{\xi }(x) + \partial _{h} {(\partial _{t} f'(u)) \xi (x-h/2)} + f'(u(x+h/2)) {\bar{\xi }}_t(x)\\&\quad \quad ~ \!+ \partial _{h} {f'(u)}\xi _t(x-h/2) + \partial _{h} {(\partial _{t} f'(u){\eta })} + \partial _{h} {(f'(u)\eta _t)} - R_1(u(x+h/2)) \partial _{h} {(\partial _{t} e^2)} \\&\quad \quad ~ \!- \partial _{h} {R_1} \partial _{t} e^2(x- h/2) - \partial _{t} R_1(u(x+h/2)) \partial _{h} {e^2} - \partial _{h} {(\partial _{t} R_1) e^2(x-h/2)} \\&\quad \triangleq \pi _1 + \cdots + \pi _{10}. \end{aligned}$$

This allows the right side of (7.13) to be written as

7.14 $$\begin{aligned} {{\mathcal {H}}} (\partial _{t} \partial _{h} {(f(u) - f(u_h))},{\bar{\xi }}_t) = \Pi _1 + \cdots + \Pi _{10} \end{aligned}$$

with $\Pi _i = {{\mathcal {H}}} (\pi _i,{\bar{\xi }}_t)$ for $i = 1, \ldots , 10$, which is estimated separately below.

By (2.11a) in Lemma 1, it is easy to show for $\Pi _1$ that

7.15a

where we have used the estimate (3.17), the relation (3.24), and also the Young’s inequality. Analogously, for $\Pi _2$ and $\Pi _4$, we apply Corollary 1 to get

7.15b $$\begin{aligned} |\Pi _2|&\le {C_{\star }}\left( {\Vert {{\bar{\xi }}_t}\Vert ^2}+ h^{-1} {\vert \![{\xi }]\!\vert }^2+ h^{2k+2}\right) , \end{aligned}$$

7.15c $$\begin{aligned} |\Pi _4|&\le {C_{\star }}\left( {\Vert {{\bar{\xi }}_t}\Vert ^2}+ {\Vert {\xi _{tt}}\Vert ^2}+ h^{-1} {\vert \![{\xi _t}]\!\vert }^2+ h^{2k+2}\right) , \end{aligned}$$

where we have also used the estimates (3.4a)–(3.4c), and the relation (3.25). A direct application of (2.11b) in Lemma 1 together with the assumption that $f'(u) \ge \delta >0$ leads to the estimate for $\Pi _3$:

7.15d

Noting that $\eta _t = u_t - P_h^- (u_t)$, we have, by Corollary 2

7.15e $$\begin{aligned} |\Pi _5|+|\Pi _6| \le {C_{\star }}h^{k+1}{\Vert {{\bar{\xi }}_t}\Vert }. \end{aligned}$$

By an analysis similar to that in the proof of (3.13), we get

7.15f $$\begin{aligned} |\Pi _7|&\le C ({\Vert {\xi _t}\Vert }+ {\Vert {{\bar{\xi }}_t}\Vert }+ h^{k+1}) {\Vert {{\bar{\xi }}_t}\Vert },\end{aligned}$$

7.15g $$\begin{aligned} |\Pi _8|&\le C ({\Vert {\xi _t}\Vert }+ h^{k+1}){\Vert {{\bar{\xi }}_t}\Vert }, \end{aligned}$$

7.15h $$\begin{aligned} |\Pi _9|&\le C ({\Vert {\bar{\xi }}\Vert }+ h^{k+1}){\Vert {{\bar{\xi }}_t}\Vert }, \end{aligned}$$

7.15i $$\begin{aligned} |\Pi _{10}|&\le C ({\Vert {\xi }\Vert }+ h^{k+1}){\Vert {{\bar{\xi }}_t}\Vert }. \end{aligned}$$

Collecting the estimates (7.15a)–(7.15i) into (7.13) and (7.14), we get, after a straightforward application of Cauchy–Schwarz inequality and Young’s inequality, that

where we have used the estimates (3.4a), (3.4c), (3.17) and (3.26) in the last step. Now, we integrate the above inequality with respect to time between 0 and *T* and combine with the initial error estimate (7.12) to obtain

By the estimates (3.4a), (3.4c) and (3.17) again, we arrive at

Finally, Gronwall’s inequality gives

7.16

This completes the proof of Lemma 8.

### The proof of Lemma 9

We will only give the proof for $|a(x)| \ge 0$, for example $a(x) > 0$; the general case follows by using linear linearization of *a*(*x*) at $x_j$ in each cell $I_j$ and the fact that $|a(x) - a(x_{j})| \le C h$. For $a(x) > 0$, by Galerkin orthogonality, we have the error equation

$$\begin{aligned} \left( {e_t},v_h\right) = {{\mathcal {H}}} (a \,e,v_h), \end{aligned}$$

which holds for any $v_h \in { V_{h}^\alpha }$. If we now take *m*-th order time derivative of the above equation and let $v_h = \partial _{t}^{m}\xi$ with $\xi = P_h^- u - u_h$, we arrive at

7.17 $$\begin{aligned} \frac{1}{2}\frac{d}{dt}{\Vert {\partial _{t}^{m}\xi }\Vert ^2} + \left( \partial _{t}^{m+1}{\eta },\partial _{t}^{m}\xi \right) = {{\mathcal {H}}} (a \, \partial _{t}^{m}\xi ,\partial _{t}^{m}\xi ) + {{\mathcal {H}}} (a \, \partial _{t}^{m}{\eta },\partial _{t}^{m}\xi ). \end{aligned}$$

By (2.11b) and the assumption that $a(x)>0$, we get

It follows from Lemma 2 that

$$\begin{aligned} {{\mathcal {H}}} (a \partial _{t}^{m}{\eta },\partial _{t}^{m}\xi ) \le C h^{k+1}{\Vert {\partial _{t}^{m}\xi }\Vert }. \end{aligned}$$

Inserting above two estimates into (7.17), we have

$$\begin{aligned} \frac{1}{2}\frac{d}{dt}{\Vert {\partial _{t}^{m}\xi }\Vert ^2} \le C {\Vert {\partial _{t}^{m}\xi }\Vert ^2} + C h^{2k+2}, \end{aligned}$$

where we have used the approximation error estimates (2.10a) and Young’s inequality. For the initial error estimate, we use an analysis similar to that in the proof of (7.6) to get

$$\begin{aligned} {\Vert {\partial _{t}^{m}\xi (0)}\Vert } \le C h^{k+1}. \end{aligned}$$

To complete the proof of Lemma 9, we need only to combine above two estimates and use Gronwall’s inequality.
